# Lymphatic vessel: Origin, heterogeneity, biological functions and therapeutic targets

**DOI:** 10.1038/s41392-023-01723-x

**Published:** 2024-01-03

**Authors:** Zhaoliang Hu, Xushi Zhao, Zhonghua Wu, Bicheng Qu, Minxian Yuan, Yanan Xing, Yongxi Song, Zhenning Wang

**Affiliations:** Department of Surgical Oncology and General Surgery, The First Hospital of China Medical University; Key Laboratory of Precision Diagnosis and Treatment of Gastrointestinal Tumors (China Medical University), Ministry of Education, 155 North Nanjing Street, Heping District, Shenyang, 110001 China

**Keywords:** Lymphangiogenesis, Metastasis

## Abstract

Lymphatic vessels, comprising the secondary circulatory system in human body, play a multifaceted role in maintaining homeostasis among various tissues and organs. They are tasked with a serious of responsibilities, including the regulation of lymph absorption and transport, the orchestration of immune surveillance and responses. Lymphatic vessel development undergoes a series of sophisticated regulatory signaling pathways governing heterogeneous-origin cell populations stepwise to assemble into the highly specialized lymphatic vessel networks. Lymphangiogenesis, as defined by new lymphatic vessels sprouting from preexisting lymphatic vessels/embryonic veins, is the main developmental mechanism underlying the formation and expansion of lymphatic vessel networks in an embryo. However, abnormal lymphangiogenesis could be observed in many pathological conditions and has a close relationship with the development and progression of various diseases. Mechanistic studies have revealed a set of lymphangiogenic factors and cascades that may serve as the potential targets for regulating abnormal lymphangiogenesis, to further modulate the progression of diseases. Actually, an increasing number of clinical trials have demonstrated the promising interventions and showed the feasibility of currently available treatments for future clinical translation. Targeting lymphangiogenic promoters or inhibitors not only directly regulates abnormal lymphangiogenesis, but improves the efficacy of diverse treatments. In conclusion, we present a comprehensive overview of lymphatic vessel development and physiological functions, and describe the critical involvement of abnormal lymphangiogenesis in multiple diseases. Moreover, we summarize the targeting therapeutic values of abnormal lymphangiogenesis, providing novel perspectives for treatment strategy of multiple human diseases.

## Introduction

The lymphatic system is a vital component of the circulatory system and plays a crucial role in maintaining fluid homeostasis, lipid absorption and the immune response in the body. Comprising a vast network of lymphatic vessels, this intricate lymphatic system is a conduit for the transportation of lymph fluid, immune cells, and various macromolecules. Lymphatic vessels are lined with lymphatic endothelial cells (LECs) with distinct structures and functions in the hierarchical lymphatic network.^1^ Additionally, it is becoming increasingly clear that adult lymphatic vessels exposed to different organ-specific environments acquire distinct characteristics and in turn execute multiple tissue-specific functions. Abnormal lymphangiogenesis can be induced under pathological conditions, where it becomes an active player in the pathogenesis of several diseases, such as lymphedema, obesity and cancer.^2^ Recently, molecular and genetic techniques have propelled the identification of potential therapeutic targets to modulate lymphangiogenesis. In this review, we provide a comprehensive summary of lymphatic vessels, addressing their origin, heterogeneity, biological functions, and related therapeutic targets.

## Historical research and milestone events of lymphangiogenesis

### Milestone events of lymphatic vessel anatomy and function

The anatomy and function of lymphatic vessels have been investigated for more than 2000 years, and many researchers have contributed numerous landmark discoveries that have led to the gradual clarification of the structure and physiological function of lymphatic vessels.^3,4^ The earliest record of the lymphatic system is the finding of lymph nodes. In the 5th century B.C., Hippocrates first coined the term *chylos* (chyle) and observed *lymphatic glands* (lymph nodes) located in the armpits, near the ears, around jugular vessels, and spread through diverse locations in the abdominal cavity. Moreover, Hippocrates described a milky fluid, termed *ichor* (lymph fluid), flowing in the lumen of some vessels. Then, Aristotle (384–322 B.C.) identified some unique fibers between blood vessels and nerves that were filled with fluid, thereby first describing the anatomy of general lymphatic vessels.^5^ As interest increased, lymphatic vessels were clearly described in a variety of tissues. Herophilus (335–280 B.C.) and Erasistratus (304–250 B.C.) successively found chyliferous vessels inside the mesentery.^6,7^ In 1536, Massa (1485–1569) found some vessels (lymphatics) in fat tissue near kidneys (renal lymphatic vessels).^8^ Eustachi (1520–1574) found the *vena alba thoracis* (thoracic duct) during horse dissection but failed to identify the extended structure and function.^9^ In 1627, Gaspare Aselli found the *venae albae aut lacteae* (the gut lacteal, a specialized capillary lymphatics with a blind end that absorbs chylomicron from intestinal villi) in a dog in 1627.^10^ Immediately afterward, in 1628, Fabrice de Peiresc described lacteals in the human body.^11^

Then, researchers went beyond anatomical studies and began to investigate the role of lymphatic vessels in lymph drainage. Jean Pecquet (1624–1674) described the *cisterna chyli* (reservoir of the chyle) and thoracic duct and explained that lymph drained into the left subclavian vein via the thoracic duct not the liver.^12^ At approximately the same time, another lymphatic vessel that converge with the thoracic duct was also described. Olaus Rudbeck (1630–1702) found *ducti hepatici aquosi* (watery hepatic ducts), now known as hepatic lymphatic vessels. Rudbeck also found cardiac, renal, pulmonary, and peripheral lymphatic vessels in 1653.^13,14^ In the same year, another researcher, Thomas Bartholin (1616–1680), coined the term *vasae lymphatica* (lymphatics or lymphatic vessels) to describe the ducts conveying lymph fluid and distinguished mesenteric lymphatic vessels from hepatic lymphatic vessels, confirming that the lymph fluid from two different sources flows into the thoracic duct.^15^ Niels Stensen (1638–1686) specifically described that both the thoracic duct and left jugular lymphatic vessel delivered lymph into the angle between the duct of the left subclavian and internal jugular veins.^4^ Simultaneously, in 1675, Stensen also discovered cervical lymphatic vessels and lymph nodes.^4^ Subsequently, in 1701, Frederik Ruysch explored the morphology and function of lymphatic valves, which ensure the unidirectionality of lymph flow.^16^ Paolo Mascagni described lymphatic vessels in the human dura mater (meningeal lymphatic vessels) and lymph node-related lymphatic vessels in 1787.^17,18^

**Fig. 1:**
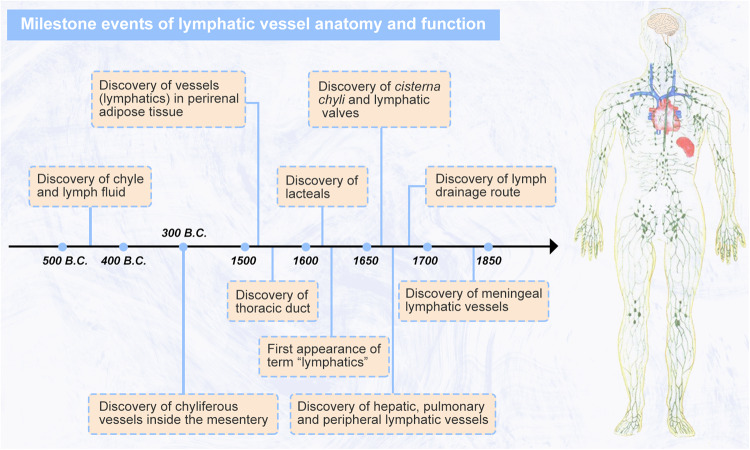
Milestone events of lymphatic vessel anatomy and function. Since the first discovery of chyle and lymph fluid at 5th B.C., some milestone findings have gradually revealed lymphatic vessel network and function in mammals. Created with Adobe Illustrator

Despite the lack of advanced microscopy imaging techniques and specific lymphatic markers to stain, the striking findings and continued exploration laid the foundation for modern anatomy and knowledge about the function of lymphatic vessels. Herein, we clearly present the early research events and critical timepoints of lymphatic vessel discoveries in Fig. 1.

### Milestone events of lymphatic vessel origins and development

**Fig. 2:**
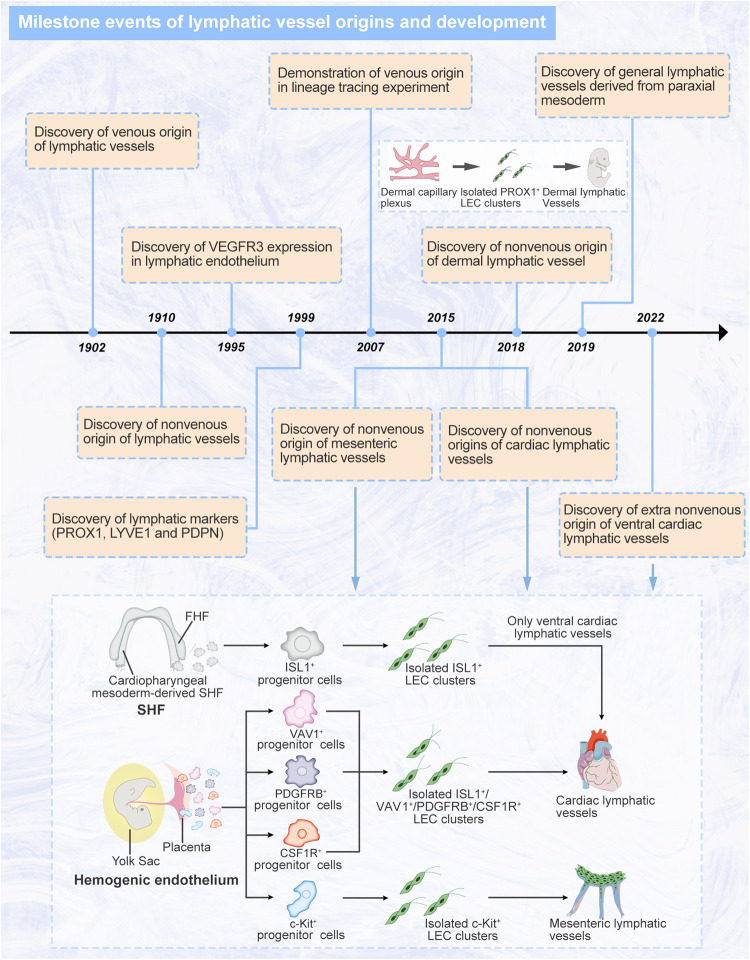
Milestone events of lymphatic vessels origins and development. In modern research (from 1902 to now), numerous researchers have gradually discovered the diverse origins and molecules of the lymphatic vessel development. These studies have doubtless initiated the understanding of heterogeneous development processes and regulatory mechanism of lymphatic vessels. VEGFR3 vascular endothelial growth factor receptor 3, PROX1 prospero homeobox protein 1, LYVE1 lymphatic vessel endothelial hyaluronan receptor 1, PDPN podoplanin, LEC lymphatic endothelial cell, FHF first heart field, SHF second heart field, ISL1 Islet 1, VAV1 vav guanine nucleotide exchange factor 1, PDGFRB platelet-derived growth factor receptor B, CSF1R colony-stimulating factor 1 receptor. Created with Adobe Illustrator

Early studies revealed the anatomy and function of mature lymphatic vessels, while modern research has been focused on the embryonic events of lymphatic vessel formations comprising origins and development (the specific events and timepoints of their discoveries are presented in Fig. 2). In 1902, Florence Sabin discovered the origin and development of lymphatic vessels by injecting India ink into pig embryos and then proposed the venous-origin theory of lymphatic vessels, which suggests that LECs sprout from veins and form the lymph sacs involved in general lymphatic vessel development.^19^ This researcher was the first to visualize the origin of lymphatic vessels and the development process. In contrast, in 1910, Huntington, McClure, and Kampmeier separately proposed a nonvenous theory of lymphatic vessel origin, suggesting that lymphatic vessels concentrically grow from isolated mesenchymal lymphangioblasts, coalesce into lymphatic sacs, and then connect to the venous system.^20^ These origin theories have been explored and verified for nearly a century. With the application of lineage tracing and other techniques in different animal models, the diversity of lymphatic vessel origins has been gradually revealed.

Martin Schneider and Annelii Ny supported the theory that lymphatic vessels originate from nonvenous cells and found that lymphangioblasts participate in the development of lymphatic vessels in avian wing bud and *Xenopus laevis* tadpole models in 1999 and 2005, respectively.^21,22^ The zebrafish is an optimal vertebrate model owing to its fast reproduction rate and ability of researchers to visualize their internal structures. In 2006, Axel M Küchler and Karina Yaniv respectively reported development and regulatory factors of lymphatic vessels in zebrafish models and supported the theory that lymphatic vessels are derived from embryonic veins.^23,24^ Additionally, mice are a proper mammalian models and have been used to show the process of lymphatic vessel development in different embryonic stages. In 2007, Sathish Srinivasan demonstrated that lymphatic vessels originated from venous endothelial cells (VeECs) by establishing prospero homeobox protein 1 (PROX1)-creERT2 model mice, PROX1 mainly drives the direct reprogramming of VeECs that form the functional lymphatic system that lasts a lifetime.^25,26^ René Hägerling showed the detailed process of embryonic lymphangiogenesis by applying ultramicroscopy to the study of whole-mount immunostained murine embryos in 2013.^27^ Hägerling found that lymphatic endothelial progenitor cells were selectively located on the dorsolateral wall of the cardinal vein, indicating that specific VeECs participate in lymphangiogenesis. Using lineage tracing and whole-mount immunostaining on different embryonic days, Oliver A. Stone found that the general lymphatic endothelium was derived mainly from paraxial mesoderm not lateral plate mesoderm. He further indicated that paraxial mesoderm-derived PAX3^+^ VeECs transdifferentiated into PROX1^+^ lymphatic endothelial progenitor cells in the cardinal veins and intersomitic veins. PAX3 is a marker for identifying myogenic progenitors in a subset of the somatic paraxial mesoderm, which could differentiate into muscular tissue and endocardium.^28^ Genetic lineage tracing in the past decade revealed the nonvenous cellular origins of a variety of organ-specific lymphatic vessels. Ines Martinez-Corral identified an isolated cell population involved in the formation of murine lumbar and dorsal midline dermal lymphatic vessels, showing another origin of dermal lymphatic vessels in 2015.^29^ Three years later, by applying genetic lineage tracing, Cathy Pichol-Thievend demonstrated that this progenitor cell population originated from a blood capillary plexus.^30^ Moreover, in 2015, Lukas Stanczuk found a population of hemogenic endothelium-derived c-Kit^+^ progenitor cells that may be involved in mesenteric lymphatic vessel development was discovered.^31^ Cardiac lymphatic vessels have also been shown to be derived from diverse cellular sources. In 2015, by using multiple Cre-loxp system-based lineage tracing, Linda Klotz determined that multiple populations of hemogenic endothelium-derived VAV1^+^/PDGFRB^+^/CSF1R^+^ progenitor cell contributed to cardiac lymphatic vessel development.^32^ Additionally, Kazuaki Maruyama and Ghislaine Lioux separately in 2019 and 2020 identified a population of second heart field-derived ISL1^+^ progenitor cells that participated in the formation of ventral cardiac lymphatic vessels.^33,34^ Two years later, Kazuaki Maruyama found that these ISL1^+^ progenitor cells originating from the cardiopharyngeal mesoderm differentiated into craniofacial and cardiac LECs.^35^

In the 1990s, a large number of studies on the regulatory factors and markers of lymphatic vessel have begun to emerge in the research field. In 1995, Kaipainen et al. discovered FLT4 gene (encoding vascular endothelial growth factor receptor 3, VEGFR3) becomes restricted in lymphatic endothelium during embryonic development.^36^ Subsequently, Kari Alitalo and Vladimir Joukov were the first researchers to isolate the ligand vascular endothelial growth factor C (VEGFC), the ligand for VEGFR2/VEGFR3 and a key factor in regulating LECs sprouting by activating VEGFR3 signaling, as proven by follow-up studies.^37,38^ In 1999, Guillermo Oliver and Jeffrey T Wigle demonstrated that PROX1 is the master regulator of lymphatic fate and regulates the expression of other transcription factors during embryonic lymphangiogenesis.^39^ At the same year, Silvana Breiteneder-Geleff discovered a transmembrane glycoprotein in podocytes, podoplanin (PDPN, encoded by T1α), which was specifically expressed in the endothelium of capillary lymphatics and was, therefore, the first lymphatic marker for immunolocalization and distinguishing the blood from lymphatic vessels.^40^ Simultaneously, Suneale Banerji determined that lymphatic vessel endothelial hyaluronan receptor 1 (LYVE1) is another specific lymphatic marker.^41^

These historical studies and milestone events reveal the diversity and heterogeneity of lymphatic vessel anatomy, function, and development and demonstrate the enthusiasm of these researchers for lymphatic vessel research.

## Lymphatic vessel development and related regulatory signaling pathways

Lymphatic vessels in embryos undergo a stepwise developmental process, including lymphatic endothelial progenitor cell specification, LEC migration, and lymphatic vessel assembly and maturation.^42^ Lymphatic vessel development involves lymphangiogenesis and lymphvasculogenesis. Lymphangiogenesis, sprouting from preexisting lymphatic vessels/embryonic veins to form new lymphatic vessels, is the main developmental mechanism underlying the formation and expansion of lymphatic networks in an embryo.^43,44^ Another mechanism, termed lymphvasculogenesis, is the process by which various populations of nonvenous cells de novo express lymphatic markers and directly incorporated into new lymphatic vessels.^43,45^ Lymphatic vessel development is a highly conserved process across multiple species and relies on a stepwise and precise regulatory program.^46^ Currently available studies only observe localized lymphatic vessel anatomy in human fetuses, lacking dynamic developmental process.^47–49^ However, few studies have recorded the complete process of lymphatic vessel development. Due to ethical issues in using human fetuses, most studies of lymphatic vessel development and function have been carried out with other vertebrate animals, such as mice and zebrafish, and with contributions from these studies, the regulatory map of lymphatic vessel development is gradually being completed.^50,51^

### Murine lymphatic vessel development and regulatory signaling pathways

#### General lymphatic vessel development and signaling pathways

Based on diverse studies with murine embryos, the timing and processes of lymphatic vessel development have been revealed.^27^ On approximately embryonic day 9.5–10.5 (E9.5–E10.5), a portion of VeECs located in the cardinal vein and intersomitic veins gradually transdifferentiate into lymphatic endothelial progenitor cells, which is the initial event in lymphatic vessel development.^52^ SRY-box transcription factor 18 (SOX18) and the chicken ovalbumin upstream promoter transcription factor 2 (COUP-TFII, encoded by NR2F2) are initially expressed in VeECs, which could synergistically activate PROX1 expression.^53,54^ Furthermore, COUP-TFII cooperates with PROX1 to upregulate the expression of VEGFR3, Neuropilin 2 (NRP2, a coreceptor with VEGFR3), and LYVE1.^55,56^ In turn, activated VEGFR3 contributes to the consistent expression of PROX1. The regulatory feedback loop further promotes lymphatic phenotype differentiation.^57^

At E10.5-E15.5, VEGFR3-expressing lymphatic endothelial progenitor cells sprout and form lymph sacs in response to extracellular VEGFC signaling, which triggers tip cells to leave veins and autonomously enter the surrounding mesenchyme.^58^ During this period, lymphatic endothelial progenitor cells differentiate into LECs and acquire migration and tube formation abilities. VEGFC is necessary and sufficient for prompting lymphatic endothelial progenitor cell budding and directed migration.^38^ At this stage, collagen and calcium binding EGF domains 1 (CCBE1) and a disintegrin and metallopeptidase with thrombospondin motifs 3 (ADAMTS3) are essential for the proteolytic cleavage of the active form of VEGFC.^59,60^ Moreover, NRP2 and VEGFR3 can jointly respond to VEGFC binding to regulate LEC migration.^56^ In addition, fibroblast growth factor (FGF), Adrenomedullin, and Hippo signaling play complementary roles in LEC proliferation and migration.^61–63^ In addition to biochemical pathways, mechanical force can activate lymphangiogenesis in this stage. The expression of the zinc-finger transcription factor GATA-binding protein 2 (GATA2) enhances VEGFR3 signaling in response to changes in tissue stiffness.^64^ Moreover, increased fluid volume could stimulate β1 integrin-mediated VEGFR3 signaling.^65^ Subsequently, lymph sacs separate from the cardinal vein via the action of platelet aggregation in response to c-type lectin-like receptor 2/PDPN signaling.^66^ Interestingly, platelet also maintain the homeostasis of lymphovenous valves.^67^ Lymphovenous valves, as the only connections between the blood and lymph circulatory systems, which have been gradually characterized, maintain unidirectional lymph drainage into veins. Especially, VEGFC activates the expression of YAP and TAZ to maintain PROX1 expression, which promote the lymphovenous valves and lymphatic valves development.^68^

**Fig. 3:**
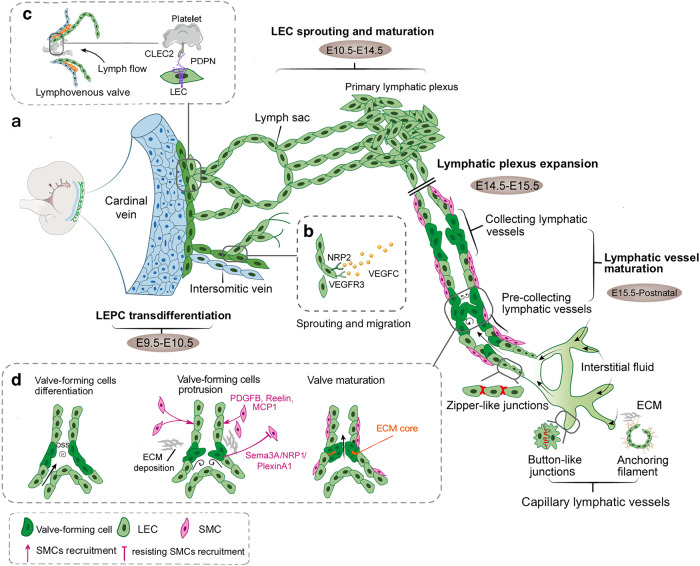
The schematic diagram of embryonic lymphatic vessel development. **a** Beginning at E9.5, VeECs, located at CV and ISVs, transdifferentiate into LEPCs. During E10.5-E15.5, the lymphatic plexus continues to sprouting and migrating, and expanding the primary lymphatic vessel network. Continuing from E15.5 until the early postnatal stage, the primary lymphatic plexus undergoes the maturation events to remodel into the hierarchical lymphatic vessels, comprising of capillary lymphatic vessels, pre-collecting lymphatic vessels, and collecting lymphatic vessels. Capillary lymphatic vessels sense interstitial pressure changes by anchoring filaments to control the opening of button-like junctions. The formation of pre-collecting and collecting lymphatic vessels requires for lymphatic valves morphogenesis and SMCs recruitment to drive lymph drainage; **b** At E10.5, upregulated VEGFR3 and NRP2 mediate LEPCs sprouting and LECs migration in response to VEGFC stimulation. VEGFC/VEGFR3 is an irreplaceable signaling regulates lymphatic vessel development; **c** The lymphovenous valve serves as the only connection of the lymphatic-venous system to prevent blood backflow. Platelet dynamically regulated lymphovenous hemostasis via interacting with LECs to activate CLEC2/PDPN signaling pathway to maintain platelet aggregation; **d** Under the stimulation of OSS, the differentiation of valve-forming cells prepares to proliferation, elongation, and protrusion. Moreover, ECM deposition and selective SMCs coverage further promote lymphatic vessel maturation. Ex embryonic day x, VeECs venous endothelial cells, CV cardinal vessel, ISVs intersomitic veins, LECs lymphatic endothelial cells, LEPCs lymphatic endothelial progenitor cells, VEGFC vascular endothelial growth factor C, VEGFR3 vascular endothelial growth factor receptor 3, NRP1/2 neuropilin 1/2, CLEC2 c-type lectin-like receptor 2, PDPN podoplanin, OSS oscillatory shear stress, MCP1 monocyte chemotactic protein 1, PDGFB platelet-derived growth factor B, ECM extracellular matrix, SMCs smooth muscle cells. Created with Adobe Illustrator

From E15.5 to the early postnatal period, the primary lymphatic plexus gradually remolds into hierarchical lymphatic vessels with different features, and these vessels are classified into capillary lymphatic vessels, pre-collecting lymphatic vessels, and collecting lymphatic vessels. The transition of intercellular junctions and changes in cell morphology are fundamental to the initial functionality of lymphatic vessels (described “Capillary lymphatics”). Additionally, the maturation of collecting lymphatic vessels involves lymphatic valve morphogenesis and smooth muscle cell recruitment, which are the structural foundations for lymph transport.^69^ The constant shear stress caused by lymph flow can stimulate the LEC-expressed mechanosensory receptors, such as PECAM, VE-cadherin, PIEZO1, β1 integrin, VEGFR2, and VEGFR3.^70^ Downstream mechanotransduction signaling maintains the expression of key transcription factors, such as PROX1, GATA2, forkhead box P2 (FOXP2), and forkhead box C1/2 (FOXC1/2), which manipulate lymphatic valve formation.^71–73^ In addition to lymphatic valves, collecting lymphatic vessels transport lymph fluid by the action of coverage of smooth muscle cells. Smooth muscle cell recruitment is regulated by the expression of platelet-derived growth factor B (PDGFB), Reelin and MCP1 in LECs.^74,75^ Moreover, FOXC2 and Angiopoietin 2 (ANG2) could activate downstream signaling to maintain the normal pattern of smooth muscle cell coverage of vessels.^73,76^ In contrast, the Sema3A/NRP1/PlexinA1 axis prevents smooth muscle cells from covering valve-forming endothelial cells.^77^ We depict the developmental process of general lymphatic vessels in Fig. 3.

#### Organ-specific lymphatic vessel development and signaling pathways

**Fig. 4:**
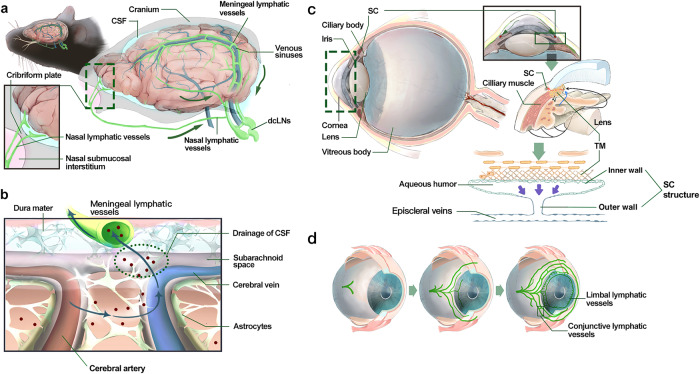
The lymphatic vessels in meninge and eyes. **a** The meningeal lymphatic vessels are mainly located at the dural region abut to the cranium, and developing along the cerebral vessels and nerves. Meningeal lymphatic vessels enter the nasal submucosal interstitium traveling through cribriform plate and form nasal lymphatic vessels extracranially, partly participating in the extracranial CSF drainage; **b** The meningeal lymphatic vessels exchange CSF with glymphatic system at subarachnoid space and eventually drain it into dcLNs; **c** SC abuts juxtacanalicular region of the TM, consisting of inner and outer wall constituted by heterogeneous endothelial cells. The inner wall could sense flow and transport aqueous humor into the SC and further drain to downstream episcleral veins; **d** The ocular surface lymphatic vessels originate from the nasal canthus and encircle laterally along the corneal limbus and the bulbar conjunctiva. CSF cerebrospinal fluid, dcLNs deep cervical lymph nodes, SC Schlemm’s canal, TM trabecular meshwork. Created with Adobe Illustrator

**Fig. 5:**
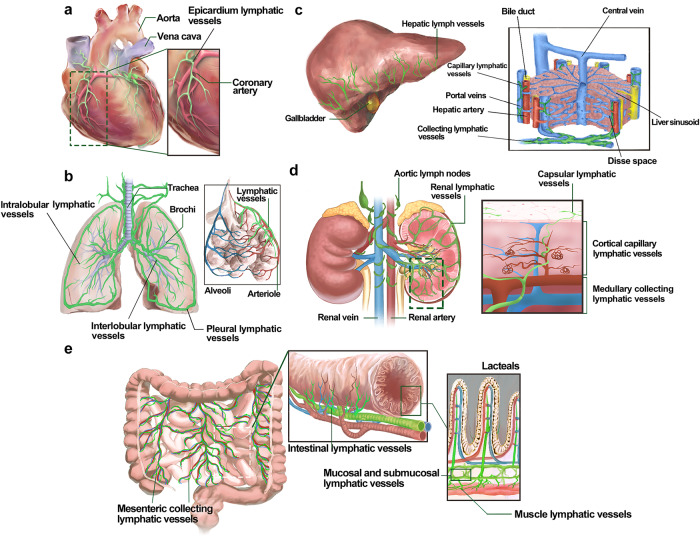
The lymphatic vessels in heart, lung, liver, kidney, and intestine. **a** Cardiac lymphatic vessels develop from the extracardiac region and follow the basal-to-tip manner along the developing coronary arteries to the ventricles; **b** Pulmonary lymphatic vessels consists of interlobular, intralobular and pleural lymphatic vessels, and develop surround airway, blood vessels and developing alveoli; **c** Capillary lymphatic vessels of the portal tract region mainly absorb the lymph secreted into the Disse space and eventually drain through collecting lymphatic vessels into thoracic duct; **d** Capsular lymphatic vessels are located near the renal surface. Cortical capillary lymphatic vessels accompany the renal tubules, glomeruli and small arteries and run along the medullary collecting lymphatic network and are finally excluded from the kidney via the hilar lymphatic vessels; **e** The intestinal lymphatic vessels consist of mesenteric collecting lymphatic vessels, mucosal, submucosal, muscle lymphatic vessels, and lacteals. Created with Adobe Illustrator

**Fig. 6:**
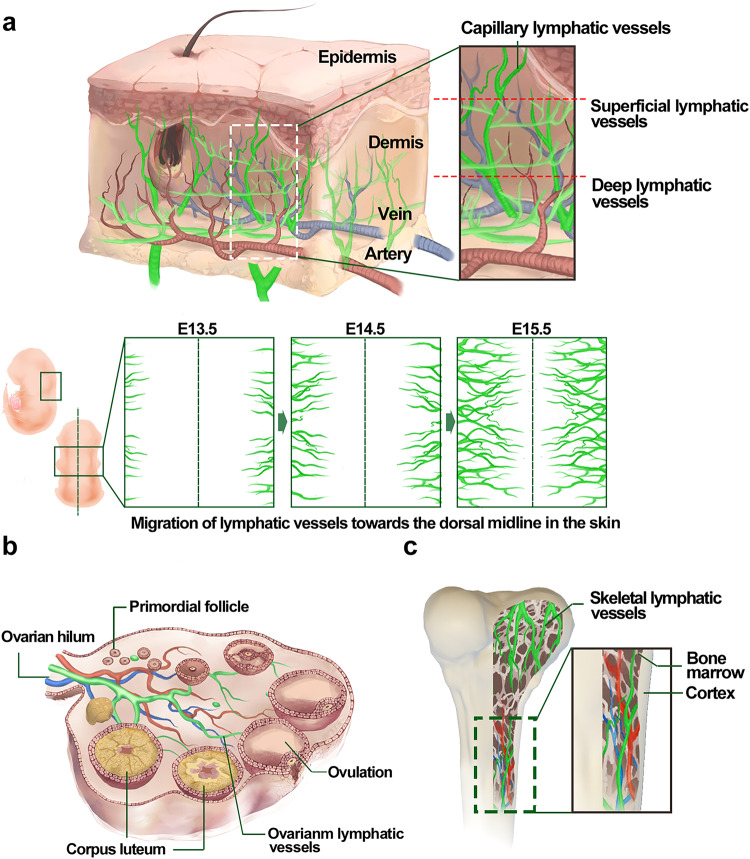
The lymphatic vessels in skin, ovary, and bone. **a** Skin lymphatic vessels, consisting of superficial and deep lymphatic vessel networks, are mainly located in the dermis and partly accompanied by dermal blood vessels. During E13.5 to E15.5, the primitive lymphatic plexus of the dorsally cervical region of skin begins to develop from the bilateral sides toward the midline of the back; **b** Ovarian lymphatic vessels develop along established blood vessels in the interstitium, which happened during the period of the first wave of follicular development; **c** Skeletal lymphatic vessels could develop at the sternum, femur, and tibia. And this part shows the skeletal lymphatic vessels go along the bone marrow of the long bones. Ex embryonic day x. Created with Adobe Illustrator

Lymphatic vessels undergo a general development process mediated through biochemical and mechanical signaling pathways and gradually form a specialized lymphatic vessel network. To adapt to the physiological needs of different organs, lymphatic vessel development involves heterogeneous processes and responses to lymphangiogenic signaling.^78^ Therefore, we summarize the development process of organ-specific lymphatic vessels and regulatory signaling in Table 1. We also depict schematic diagrams of the murine organ-specific lymphatic vessel anatomy in Figs. 4–6.

**Table 1:** Organ-specific lymphatic vessel development and regulatory signaling pathways in mouse

Organ-specific lymphatic vessels	Developmental stages and events	Regulatory signaling pathways	Reference
Meningeal lymphatic vessels	P0–P28: Venous-origin lymphatic vessels start sprouting around the foramen magnum and gradually cover the most meninges of the central nervous system, and meningeal lymphatic vessel development is provided with the specific niches by dural sinuses, cerebral arteries, and central nerve bundles.	1. VEGFC/VEGFR3 signaling regulates meningeal lymphatic vessel development and maintains structural integrity. 2. Mechanical forces maintain the maturation and function of meningeal lymphatic vessels after birth, which is mediated by PLCγ2 involved in the CLEC2/PDPN signaling to control lymph flow.	^125,389–393^
Ocular lymphatic vessels	P0-P14: Ocular surface lymphatic vessels sprout from pre-existing lymphatic vessels and begin from the inner canthus, and then gradually develop into limbal and conjunctive lymphatic vessels following a nasal-to-temporal manner. P1-P17: Schlemm’s canal derived from episcleral veins follows a similar developmental manner via sprouting and interconnecting into the vessel-like structure, and then endothelial cells express PROX1 but acquire mixed cell identity with partial vascular and lymphatic markers. Subsequently, Schlemm’s canal continues maturing and possesses a luminal structure with two layers of endothelial cells.	1. VEGFC/VEGFR3 signaling mainly activates the ocular surface lymphatic vessels and Schlemm’s canal development. 2. ANG1/2 binding to TIE1/2 could enhance ocular surface lymphatic vessels and Schlemm’s canal development, meanwhile, ANG4 and SVEP1 are also involved in this regulatory pathway. 3. The shear stress-controlled transcription factor KLF4 physically binds to the first intron of the PROX1 coding sequence and induces PROX1 expression to maintain Schlemm’s canal identity and integrity.	^237,238,394–399^
Cardiac lymphatic vessels	E12.5-E14.5: Developmental cardiac lymphatic vessels sprout from both the outflow tract and sinus venous in a base-to-apex manner. E14.5-E18.5: Diverse non-venous progenitor populations participate in cardiac lymphatic vessel development via lymphvasculogenesis. P0-P15: Cardiac lymphatic vessels laterally and deeply sprout and cover most regions of the epicardium and myocardium of the postnatal heart.	1. VEGFC/VEGFR3 signaling regulates the morphogenesis of cardiac lymphatic vessels, and transcription factors TBX1 and CCBE1 could be involved in this process. 2. VE-cadherin regulates cardiac lymphatic vessel development and postnatal structural stability via mediating Adrenomedullin signaling transactivating VEGFR3 by c-Src. Additionally, Adrenomedullin signaling could regulate RAP1-mediated lymphatic endothelial junction integrity.	^284,400–404^
Pulmonary lymphatic vessels	E11.5-E14.5: LECs migrate in a proximal-to-distal manner into the developing lung lobes, and then the primary lymphatic plexus expands along the bronchovascular bundles. E18.5-P0: The pulmonary lymphatic vessel network has been widely distributed in the airways and lower respiratory bronchioles, as well as existing abut intralobular arterioles and small veins. Around birth, pulmonary lymphatic vessels rapidly function in response to the surge generation of interstitial fluid along the change of button-like junctions.	1. VEGFC/VEGFR3 signaling mediates pulmonary lymphatic vessel development from the embryonic to the neonatal period. 2. At birth, transient expression of c-JUN can induce CDH13 and ATF3 expression, remodeling the conformation of the actin cytoskeleton and initiating lymphatic vessel drainage in response to high shear stress.	^405–407^
Hepatic lymphatic vessels	P1–P7: The primary hepatic lymphatic vessels appear at large superficial portal tracts. P8–P21: The lymphatic vessels continuously sprout deeply and terminally distribute at the portal vein region, the hepatic vein region, and the hepatic capsule region.	1. Heterozygous mutations of VEGFC/VEGFR3 delay hepatic lymphatic vessel development and disrupt the lymphatic vessel structure. 2. CHD4 raises the transcriptional activity of uPAR to activate plasmin, maintaining the development and structure of hepatic lymphatic vessels and lymphovenous valves.	^408–411^
Intestinal lymphatic vessels	E12-E13: The right subcardinal veins-derived LECs outgrow ventrolaterally to form retroperitoneal lymph sacs. Subsequently, mesenteric lymphatic vessels gradually sprout from retroperitoneal lymph sacs and go along the established mesenteric blood vessels at the left region of the dorsal mesentery. E13.5: A population of the hemogenic endothelium-derived c-Kit^+^ progenitor cells incorporates into mesenteric lymphatic vessels via lymphvasculogenesis. E14.5-E15.5: The mesenteric lymphatic vessels continue expanding and forming intestinal wall lymphatic vessels, filling the submucosa along the branch trail of the arteries. E17.5-P10: The mesenteric lymphatic vessels sprout into the majority of the villus and form the mature lacteals.	1. ANG/TIE and Adrenomedullin/CALCRL/ERK signalings respectively activate downstream cascades to stimulate intestinal lymphatic vessel development. 2. VEGFR3/PI3K signaling has a selective role in regulating intestinal lymphatic vessel development dependent on different regulatory subunits, including p110α, p85α, p55α, and p50α. 3. Some negative regulators stabilize intestinal lymphatic vessel development and integrity by antagonizing VEGFC/VEGFR3 signaling, including RASA1, CCM3, and Claudin-like proteins.	^31,113,412–418^
Renal lymphatic vessels	E14.5-E15.5: Renal lymphangiogenesis sprouts from the renal hilum based on the establishment of massive vascular networks. E16.5-E18.5: The developing hilar lymphatic vessels continue remolding and extending towards the renal cortex. Meanwhile, some isolated LEC clusters participate in renal lymphatic vessel development via lymphvasculogenesis. Additionally, the ascending vasa recta, lymphatic-like vessels, are developing for lymph transport.	1. VEGFC regulates renal lymphangiogenesis and lymphvasculogenesis contributing to renal lymphatic vessel development. 2. ANG1 and ANG2 synergistically act on TIE2 for the function and stability of the ascending vasa recta.	^419,420^
Dermal lymphatic vessels	E13.5-E16.5: Dermal lymphatic vessels continuously sprout from lateral sides towards the dorsal midline in the skin via lymphangiogenesis. Meanwhile, a population of blood capillary-derived PROX1^+^ cells is involved in dermal lymphatic vessel development via lymphvasculogenesis at lumbar and dorsal midline skin.	1. CCBE1/VEGFC signaling activates lymphatic endothelial progenitor cells sprouting from veins and blood capillaries to form dermal lymphatic vessels. 2. The DLL4/NOTCH1 signaling pathway regulates VEGFR3 expression in an EphrinB2-mediated manner to affect dermal lymphatic vessel development. Additionally, NOTCH4 has a distinct influence on regulating the dermal LEC migration and lymphatic vessel branching via activating different cascades.	^30,421,422^
Ovarian lymphatic vessels	P8.5-P12.5: The ovarian lymphatic vessels sprout from the hilum of the ovary, and then continuously sprout towards the ovarian mesenchyme.	1. Ovarian lymphatic vessels develop is mediated by VEGFC/VEGFR3 signaling in an ADAMTS1-dependent manner. 2. Follicle-stimulating hormone and estradiol regulate VEGFC/D/VEGFR3-mediated regional ovarian lymphangiogenesis.	^423–425^
Skeletal lymphatic vessels	Recently, existing of skeletal lymphatic vessel in physiological conditions has been first revealed, however, the process of skeletal lymphatic vessel development is rarely documented.	VEGFC/VEGFR3 signaling prompts skeletal lymphangiogenesis.	^283^

*LEC* lymphatic endothelial cell, *VEGFC* vascular endothelial growth factor C, *VEGFR3* vascular endothelial growth factor receptor 3, *PLCγ2* phospholipase C gamma 2, *ANG* angiopoietin, *CLEC2* c-type lectin-like receptor 2, *PDPN* podoplanin, *TIE* tunica interna endothelial cell kinase, *PROX1* prospero homeobox protein 1, *CHD4* chromodomain helicase DNA binding protein 4, *uPAR* urokinase-type plasminogen activator receptor, *SVEP1* sushi von Willebrand factor type A EGF and pentraxin domain containing 1, *KLF4* KLF transcription factor 4, *TBX1* T-box 1, *CCBE1* collagen and calcium binding EGF domains 1, *CDH13* cadherin 13, *ATF3* activating transcription factor 3, *RAP1* Ras-related protein 1, *RASA1* RAS p21 protein activator 1, *CCM3* cerebral cavernous malformation 3, *DLL4* delta like canonical Notch ligand 4, *VE-cadherin* vascular endothelial-cadherin, *CALCRL* calcitonin receptor-like receptor, *ERK* extracellular signal-regulated kinase, *ADAMTS1* a disintegrin and metallopeptidase with thrombospondin motifs 1, *PI3K* phosphoinositide 3-kinase

### Zebrafish lymphatic vessel development and related signaling pathways

**Fig. 7:**
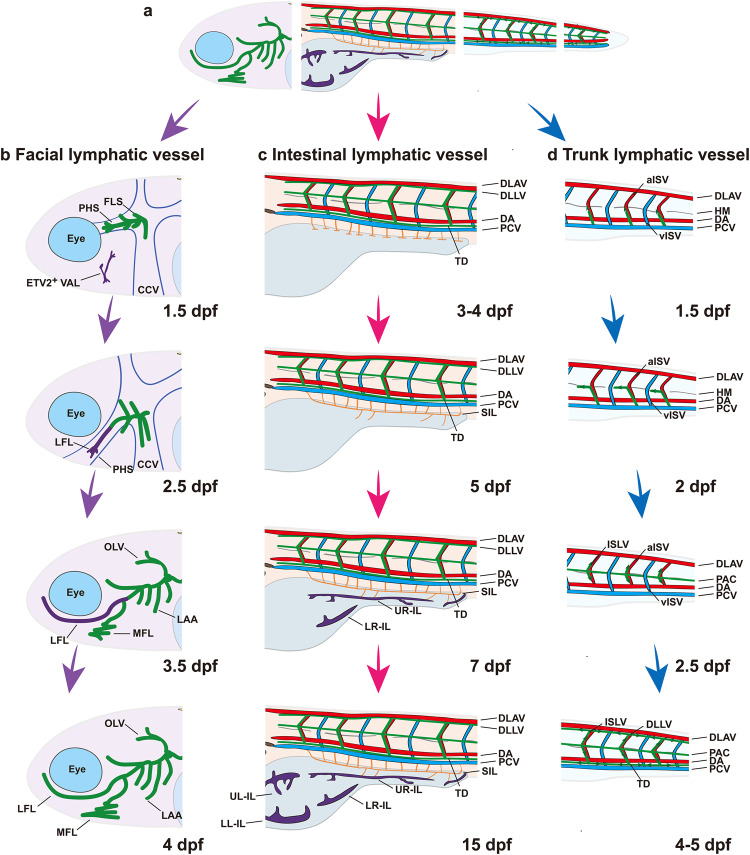
The schematic of lymphatic vessels development in zebrafish. **a** Zebrafish possess an extensive lymphatic vessel network throughout the body. Specialized lymphatic vessels development in zebrafish includes facial lymphatic vessel development (**b**), intestinal lymphatic vessel development (**c**), and trunk lymphatic vessel development (**d**); **b** The facial lymphatic vessels of zebrafish undergo a step-wise assembly from 1.5 dpf to 4 dpf. The FLS are derived from the CCV beginning to form along the PHS. Subsequently, a group of ETV2-expressing cells, known as VAL, begin to fuse with the lymphatic sprouts to form the LFL along the PHS. The LFL then begins to bud out to form a complex facial lymphatic vessels including the OLV, LAA, and MFL; **c** The development of intestinal lymphatic vessels proceeds from 3 dpf–15 dpf. At 3 dpf-4 dpf, LECs sprout from the PCV to the ventral and bilateral sides, respectively to form segmental lymphatic vessels, which subsequently interconnect to form L-SIL, R-SIL. The SILs first bud toward the right side of the abdomen along the vascular network to form UR-IL and IR-IL. Finally, the lymphatic vessel network continues to expand follow the left vascular track to form UL-IL and LL-IL and take up intestinal lymph; **d** The formation of trunk lymphatic vessels is the earliest event of embryonic lymphatic vessel development in zebrafish, budding from the PCV to form the ISLV along the trajectory of the ISVs, and subsequently sprouting ventrally and dorsally to form the DLLV and TD, respectively. Dpf day postfertilization, CCV common cardinal vein, PHS primary head sinus, FLS facial lymphatic sprouting, VAL ventral aorta lymphangioblast, LFL lateral facial lymphatic vessel, OLV otolithic lymphatic vessel, ETV2 ETS variant transcription factor 2, LAA lymphatic branchial arches, MFL medial facial lymphatic vessels, PCV posterior cardinal vein, L-SIL the left supraintestinal vessel, R-SIL the right supraintestinal vessels, UR-IL upper-right intestinal lymphatics, LR-IL lower-right intestinal lymphatics, UL-IL upper-left intestinal lymphatics, LL-IL lower-left intestinal lymphatics, ISLV intersegmental lymphatic vessel, aISV arterial intersegmental vessels, vISV venous intersegmental vessel, PAC parachordal line, DA dorsal aorta, DLAV dorsal longitudinal anastomotic vessel, HM horizontal myoseptum, DLLV dorsal longitudinal lymphatic vessel, TD thoracic duct. Created with Adobe Illustrator

Because of their relatively transparent body, large number of progeny, and short developmental cycle, zebrafish have become an optimal animal model for observing the dynamic development of lymphatic vessels.^79,80^ Through the combined application of high-resolution imaging techniques and identification of lymphatic markers, the heterogeneous processes underlying lymphatic vessel development in zebrafish have been gradually elucidated in the past decade.^79^ Although the main lymphangiogenic signaling network is conserved across mammals, lymphatic vessel development is regulated by specific molecules and signaling pathways in zebrafish. Therefore, we summarize the heterogeneous development processes of lymphatic vessels (Fig. 7) and the significant molecules and signaling pathways in zebrafish (Table 2).

**Table 2:** Molecules and related signaling pathways of lymphatic vessel development in zebrafish

Molecules	Roles and effects on lymphatic vessel development	Signaling pathways	Reference
PROX1	A transcription factor, which initiates LEPC transdifferentiation and continuously regulates lymphatic vessel development.	PROX1 induces LEC markers (VEGFR3, TBX1, CDH6, and CDN11b) expression and interplays with diverse downstream cascades (for example VEGFC/VEGFR3).	^426–428^
VEGFC	The ligand of VEGFR3, which regulates LEPC transdifferentiation as well as LEC sprouting and migration.	VEGFC selectively stimulates the downstream cascades of VEGFR3 in a context-dependent manner and maintains PROX1 expression of LEPC via activating MEK/ERK signaling.	^23,82^
VEGFD	The ligand of VEGFR2, which regulates LEC sprouting and migration in zebrafish heads.	VEGFD binding to VEGFR2 supplements the role of VEGFC/VEGFR3 signaling.	^429–431^
ADAMTS2/3/14	Extracellular matrix proteins, which regulate LEC migration.	ADAMTS3 and ADAMTS2/14 proteolytically process immature VEGFC to activate VEGFR3.	^432,433^
CCBE1	An extracellular matrix protein, which regulates LEC migration to enhance lymphatic vessel development.	CCBE1 cooperating with ADAMTS3 activates VEGFC/VEGFR3 signaling via proteolytic activation of immature VEGFC.	^84,432,434^
SOX18	A transcription factor, which regulates LEC sprouting and migration to enhance TD formation.	SOX18 directly interacts with VEGFC to activate the downstream cascades.	^426,435^
SOX17	A transcription factor, which negatively regulates LEPC transdifferentiation and realizes lymphatic-to-blood vascularization in zebrafish anal fin.	SOX17 negatively regulates the lymphatic identity via suppressing PROX1 expression.	^436^
HHEX	A transcription factor, which regulates LEPC transdifferentiation as well as LEC sprouting and migration.	HHEX is involved in the VEGFC/VEGFR3/PROX1 cascade to affect early lymphatic vessel development.	^437^
MAFB	A Transcription factor of the MAF family, which regulates LEC migration, sprouting, and patterns of facial lymphatic vessel development. MAFBa regulates LEPC sprouting and MAFBb patterns trunk lymphatic vessel development.	Transcription factor SOX7/18 stimulates the MAFBa/b expression, which is a downstream target of VEGFC/D signaling.	^438^
GATA2	A transcription factor, which regulates lymphatic valve formation and facial lymphatic vessel development.	Under the stimulation of shear stress, GATA2 activates downstream target genes (PROX1, FOXC1/2, NFATC1).	^439^
EFNB2	The ligand of EPHB4, which initiates lymphatic valves and lymphovenous valve morphogenesis.	EFNB2/EPHB4/RASA1 axis induces PROX1 expression to promote the differentiation of valve-forming cells via inhibition of ERK signaling.	^440^
WNT5	A member of the WNT family, which regulates LEPC transdifferentiation.	WNT5b upregulates PROX1 expression at the specialized niche within the cardinal vein via activating the canonical WNT/β-catenin signaling.	^81^
CXCL12	A chemokine ligand, which regulates the LEC directed migration to assemble trunk lymphatic vessels.	CXCL12a/b binding with CXCR4a/b regulates LEC migration activity.	^86,441^
BMP2	A member of the BMP family, which negatively regulates LEPC transdifferentiation and proliferation.	BMP2 signaling stimulates miR-31 and miR-181a expression in an SMAD-dependent manner and then reduces PROX1 expression.	^442,443^
DLL4	The ligand of NOTCH signaling, which regulates LEC sprouting and migration to promote TD and PLs development.	DLL4 activates NOTCH1b or NOTCH6 signaling.	^444^
Plexin D1	The receptor of Semaphorin 3AA/3 C, which negative regulates LEC sprouting and migration to inhibit facial lymphatic vessel formation.	Plexin D1 competitively inhibits VEGFC/VEGFR3/ERK signaling.	^445^

Regulators	Role and function in zebrafish lymphangiogenesis	Signaling pathways	Reference
Apelin	The ligand of APLNR, which patterns normal TD and PLs development and maintains LEC proliferation.	Apelin signaling selectively activates AKT1/2 phosphorylation.	^446^
RASGRP1	A member of the RASGRP family, which regulates LEC sprouting and migration.	RASGRP1 possibly affects the downstream effectors of VEGFR3 signaling to enhance the RAS/ERK signaling pathway.	^447^
PAR1	A kind of G-protein-coupled receptor, which regulates LEPC transdifferentiation.	Noncanonical MMP13b/PAR1/GNAI2a signaling pathway activates VEGFR3 expression which stimulates the phosphorylation of ERK1/2 to induce PROX1a expression in venous endothelial cells.	^448^
CD146	A cell adhesion molecule, which regulates LEC proliferation, sprouting, and migration.	CD146 respectively activates p38 kinase and ERK signaling in response to VEGFC.	^449^
SVEP1	An extracellular protein, which regulates LEC sprouting and migration to maintain the development and structure integrity of facial lymphatic vessels.	SVEP1 binds with TIE1 to activate the downstream cascades.	^450^
GRB2	An adapter protein, which regulates LEC sprouting and migration to selectively promote meningeal lymphatic vessel development.	GRB2b genetically interacts with VEGFR3 to activate the downstream cascades.	^451^
PKD1	A transmembrane protein, which regulates LEC migration and remolding later to maintain TD formation.	PKD1a partly interacts with WNT5a to regulate LEC sprouting from the horizontal myoseptum.	^452,453^
Synectin	A scaffold protein, which regulates LEC sprouting and migration to form TD.	Synectin stimulates VEGFR3 and NRP2a to activate downstream signaling.	^454^
YAP1	An effector of Hippo signaling, which stimulates LEPC proliferation and sprouting.	YAP1 is dependent on VEGFC to activate downstream signaling.	^455^
DDX21	A kind of RNA helicase, which balances ribosome biogenesis and the cell cycle to regulate LEPC sprouting and migration.	DDX21 inhibits p53 and p21 expression to enhance VEGFC-mediated lymphangiogenesis.	^456^
Netrin1a	A guidance protein, which regulates LEC sprouting and migration to form TD and PLs.	Netrin1a activates downstream DCC-mediated axon guidance cues for lymphangiogenesis.	^457^
NOVA2	A RNA-binding protein, which regulates LEPC transdifferentiation.	NOVA2 inhibits the PROX1 expression via negatively regulating the pre-mRNA splicing of the MAPK/ERK signaling.	^458^
VASH1	A kind of Carboxypeptidase, which regulates LEPC transdifferentiation and proliferation to enhance secondary sprouting for trunk lymphatic vessel development.	VASH1 mediates tubulin detyrosination to control the number of secondary sprouting cells.	^459^
miR-126	A member of miRNAs, which regulates LEC sprouting and migration.	MiR-126a induces the expression of CXCL12a and enhances chemokine signaling and VEGFR3 expression.	^460,461^
miR-204	A member of miRNAs, which regulates lymphatic vessel development.	MiR-204 acts on the transcription factor NFATC1.	^462^
miR-182	A member of miRNAs, which regulates LEC sprouting and migration to form the TD and PLs.	MiR-182 negatively regulates JUNB-mediated the FOXO1 expression.	^463^

*LEPC* lymphatic endothelial progenitor cell, *LEC* lymphatic endothelial cell, *TD* thoracic duct, *PLs* parachordal lymphangioblasts, *PROX1* prospero homeobox protein 1, *VEGF* vascular endothelial growth factor, *VEGFR* vascular endothelial growth factor receptor, *ERK* extracellular signal-regulated kinase, *MAPK* mitogen-activated protein kinase, *TIE* tunica interna endothelial cell kinase, *TBX1* T-box 1, *CDH6* cadherin 6, *ADAMTS* a disintegrin and metallopeptidase with thrombospondin motifs, *CCBE1* collagen and calcium binding EGF domains 1, *SOX* SRY-related HMG-box, *HHEX* hematopoietically expressed homeobox, *MAFB* V-maf musculoaponeurotic fibrosarcoma oncogene homolog B, *FOXC* forkhead box C, *NFATC1* nuclear factor of activated T-cells cytoplasmic 1, *GATA2* GATA-binding protein 2, *EFNB2* ephrin B2, *EPHB4* ephrin type B receptor 4, *RASA1* RAS p21 protein activator 1, *WNT5b* Wnt family member 5b, *CXCL12* C-X-C motif chemokine 12, *CXCR4* C-X-C chemokine receptor type 4, *BMP2* bone morphogenetic protein 2, *DLL4* delta like canonical Notch ligand 4, *RASGRP1* RAS guanyl releasing protein 1, *AKT* protein kinase B, *PAR1* protease-activated receptor 1, *MMP* matrix metalloproteinase, *GNAI2* G protein subunit alpha i2, *SVEP1* sushi von Willebrand factor type A EGF and pentraxin domain containing 1, *GRB2* growth factor receptor bound protein 2, *PKD1* polycystic kidney disease 1, *NRP2* neuropilin 2, *YAP1* Yes-associated protein 1, *DDX21* DEAD-box helicase 21, *NOVA2* Neuro-oncological ventral antigen 2, *VASH1* vasohibin 1, *DCC* Deleted in colorectal cancer (the Netrin1 receptor), *miRNAs* microRNAs, *FOXO1* forkhead box O1

Early lymphatic vessel development occurs during the first day 5 postfertilization (dpf). Similar to mammals, the posterior cardinal vein is the origin of lymphangiogenesis.^81^ Between hour 30 and 34 postfertilization (hpf), a population of bipotential progenitor cells (also known as lymphangioblasts) generates two kinds of endothelial cells, VeECs and PROX1-expressing lymphatic endothelial progenitor cells.^82^ In particular, VEGFC is the key factor that regulates the division of cell identity, and it also triggers PROX1 expression.^82^ Moreover, the role of PROX1 in initiating the transdifferentiation of VeECs into lymphatic endothelial progenitor cells is conserved in both zebrafish and mammals.^82^ At 1.5 dpf-2 dpf, LECs dorsally sprout toward the horizontal myoseptum, forming a population of LECs known as parachordal lymphangioblasts (or parachordal line). CCBE1/VEGFC/VEGFR3 signaling regulates LEC sprouting and migration.^83,84^

At 2.5 dpf-4 dpf, lymphatic vessel sprouts from the parachordal lymphangioblasts begin to migrate dorsally and ventrally along arterial intersegmental vessels not venous intersegmental vessels, forming intersegmental lymphatic vessels.^85,86^ Subsequently, LECs on the dorsal and ventral sides of the intersegmental vessels migrate rostrally and caudally and ultimately interconnect, forming the two trunk lymphatic vessels, namely, the dorsal longitudinal lymphatic vessel (abutting the dorsal longitudinal anastomotic vessel) and thoracic duct (abutting the posterior cardinal vein).^87,88^ We show the process of mainly trunk lymphatic vessel development in Fig. 7 and describe the related regulatory signaling pathways involved in LEC sprouting and migration in Table 2.

Between 5 dpf and 7 dpf, LECs begin to emerge from the intersegmental lymphatic vessels (the third trunk lymphatic vessel) and extend rostrally and caudally along the horizontal myoseptum to gradually form parachordal lymphatic vessels.^80^ Subsequently, parachordal lymphatic vessels continue to expand laterally during zebrafish development and form intercostal lymphatic vessels at approximately 15 dpf.^80^ Parachordal lymphatic vessels and intercostal lymphatic vessels become lateral lymphatic vessels in zebrafish. Through sophisticated signaling mechanisms, VEGFC/VEGFR3 is the key interaction that triggers the signaling cascade to activate LECs continuously. Interestingly, the homeobox transcription factor HOXC9 supports the expression of stabilin 2 to maintain the normal formation of thoracic duct and parachordal lymphangioblasts, and another homologous protein, stabilin 1, plays a similar role in promoting zebrafish lymphangiogenesis in a HOXC9-independent manner.^89^

In this developmental period, organ-specific lymphatic vessel development simultaneously occurs in the head and intestine of zebrafish.^80,90^ We depict the process of organ-specific lymphatic vessels by development stages in Fig. 7. Further studies should be conducted to verify the specific function of lymphangiogenic signaling pathways in organ-specific lymphatic vessel development and biological capacity.

## Biological functions of lymphatic vessels and related regulatory signaling pathways

### Anatomy and structure of the lymphatic vessel network

**Fig. 8:**
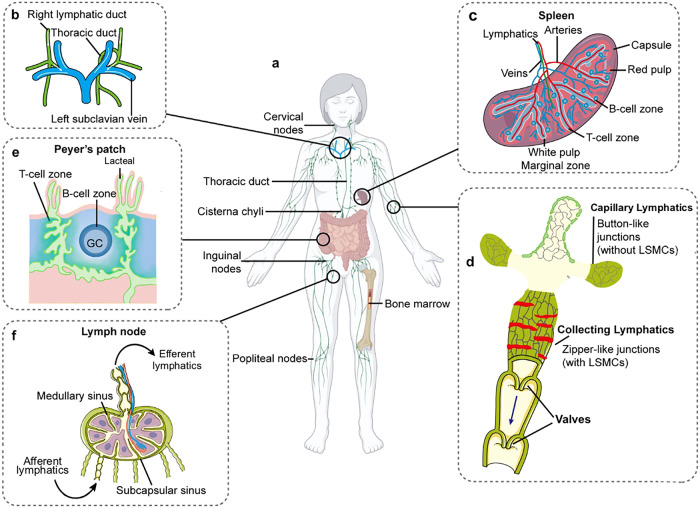
Anatomy of the lymphatic system. **a** The lymphatic system includes the primary and secondary lymphoid organs and lymphatic vessels, providing a one-way drainage route from all tissues back ultimately to the blood circulation via the great veins in the neck. In the primary lymphoid organs (bone marrow and thymus), immune cell production and maturation takes place, whereas secondary lymphoid organs (lymph nodes, spleen, and mucosa-associated lymphoid organs such as Peyer’s patch, tonsils, and adenoids) are the sites for lymphocyte activation; **b** The thoracic duct is responsible for the lymph drainage coming from most of the body with the exception of the right side of the head and neck, the right side of the thorax and the right upper limb where drain lymph primarily into the right lymphatic duct; **c**–**e** The spleen, the Peyer’s patch and lymph nodes are highly organized structures with segregated B-cell and T-cell zones to optimize the induction of adaptive immune responses; **f** The capillary lymphatics drain downstream into the collecting lymphatics. Capillary LECs are interconnected via discontinuous junctions allowing the fluid to enter the system passively. Collecting LECs present with continuous junctions. Collecting lymphatics differ from capillary lymphatics by possessing intraluminal valves, LSMCs and a continuous basement membrane. LSMCs lymphatic smooth muscle cells, LECs lymphatic endothelial cells, GC germinal center. Created with Adobe Illustrator

The lymphatic system includes primary lymphoid organs (the bone marrow and thymus), secondary lymphoid organs (the lymph nodes, spleen, and mucosal-associated lymphoid tissue), and lymphatic vessels that connect all lymphoid organs.^91^ The lymphatic vessel network is composed of capillary lymphatics, pre-collecting and collecting lymphatics.^92^ Additionally, the cell-cell junctions (button- and zipper-like junctions) are important for functionally specialized capillary and collecting lymphatics (Fig. 8).

#### Capillary lymphatics

Capillary lymphatics (also called initial lymphatics) consist of a single layer of loosely connected LECs without a continuous basement membrane and are covered by pericytes or smooth muscle cells.^93^ They are blind-ended vessels and are connected by anchoring filaments to the interstitial tissue. Overlapping endothelial cells build flap-like mini-valves, ensuring one-way interstitial fluid, macromolecule, and immune cells flow into the vessels. These capillary lymphatics in the most tissues are interconnected through discontinuous button-like junctions. Additionally, the integrity of LEC junctions is regulated by two different types of cell-cell junctions: adherens junctions and tight junctions.

The button-like junctions of the capillary lymphatics are formed by adherens junction proteins, including VE-cadherin,^94^ p120-catenin, and catenin, which bind to each other and the actin cytoskeleton. Tight junctions are formed by transmembrane proteins, such as claudin-5 and occludin, and the cytoplasmic protein ZO-1, which regulate paracellular permeability,^91^ and junctional adhesion molecule and endothelial cell-selective adhesion molecule, which are involved in leukocyte transmigration. The ANG/tunica interna endothelial cell kinase 2 (TIE2) signaling pathway is indispensable for the formation of button-like junctions within capillary lymphatics.^95^ In lacteals, VEGFC-VEGFR2/3-delta-like 4 (DLL4)-NOTCH signaling is crucial for constant regeneration and maintenance of button junctions.^96^

#### Collecting lymphatics

Capillary lymphatics drain into pre-collecting lymphatic vessels and merge with larger collecting lymphatics. Collecting lymphatic vessels contain valves that regulate the unidirectional flow of lymph with the coordinated contraction of smooth muscle cells facilitating the transport of lymph into the bloodstream.^97^ Tissue fluid transported by collecting lymphatics ultimately drains into the thoracic duct and right lymphatic duct, which discharge lymph into the common opening of the jugular and subclavian veins known as the venous angle.^98^

In collecting lymphatics, LECs are continuously connected through zipper-like junctions and are enveloped by specialized smooth muscle cells that contract to assist lymph flow.^93^ During mouse embryonic development, the transformation from continuous zipper-like junctions (formed at E12.5-E16.5) to button-like junctions begins at E17.5 and is mostly complete by postnatal day (P) 28.^99^ Therefore, zipper-like junctions are regulated in a manner similar to that of button-like junctions, however, some mechanisms that specifically regulate zipper-like junctions are described below. RhoA/ROCK signaling is essential for LEC junction formation in lacteals, lymphatic valves, and collecting vessels. Transcription factors FOXC1 and FOXC2 are required for maintaining LEC junction integrity in lymphatic valves, collecting vessels, and dermal lymphatics.^92^ Recent studies identified several other major signaling pathways that control tight junction localization and lymphatic vessel integrity, including EphrinB2/EphB4 and S1PR1.^91^

### Biological functions of lymphatic vessels

The general functions of lymphatic vessels in fluid absorption and transport, as well as immunosurveillance, are well understood. However, accumulating evidence indicates that lymphatic vessels play active and versatile roles in an organ-specific manner during homeostasis and multiple disease processes.^100^ We provide a brief overview of the novel discoveries of organ-specific functions of adult mammalian lymphatic vessels, including immunosurveillance after pathogen invasion, transport of dietary fat, and drainage of cerebrospinal fluid and aqueous humor.

#### Lymph absorption and transport

**Fig. 9:**
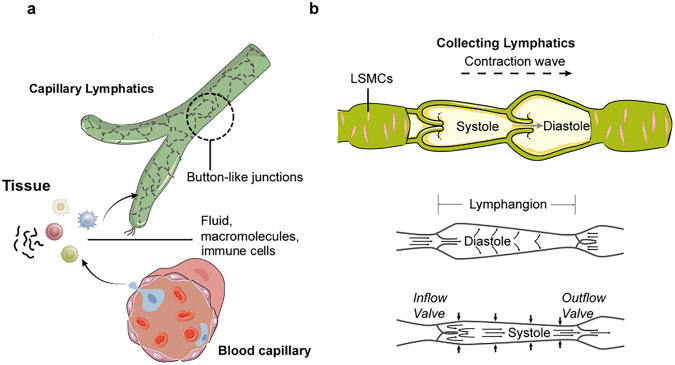
Lymph absorption and transport. **a** Capillary lymphatics comprise a single layer of loosely connected LECs lacking a continuous basement membrane and perivascular mural cells. LECs within capillary lymphatics are interconnected through discontinuous button-like junctions that facilitate the uptake of interstitial fluid, macromolecules and immune cells which are released by the blood capillary; **b** Collecting lymphatics have a period of brisk contraction (systole) and a period of relaxation (diastole) between each phasic contraction. Each lymphangion, defined as the segment between two valves, can typically exhibit systole and diastole. When a lymphangion is relaxed, the inflow (or upstream) valve will open (given sufficient inflow pressure). During systole, the phasic contraction pushes the lymph, but the inflow valve closes, so that lymph is forced forward through the outflow valve. LECs lymphatic endothelial cells. Created with Adobe Illustrator

Lymphatic vessels play crucial roles in the uptake and transport of multiple substances to maintain tissue fluid homeostasis, such as lipids, proteins, and immune cells in the body, which present diverse characteristics in different organs.^101^ Studies have suggested that both passive paracellular and active transcellular transport mechanisms may contribute to lymph absorption.^102^ Moreover, lymphatic vessels have an active role in draining excess interstitial fluid from organs and serving as conduits for immune cell trafficking to lymph nodes. The lymphatic pump undergoes phasic contractions generated by lymphatic smooth muscle cells to realize lymph transport, and lymphatic valves ensure one-way lymph transport. When upstream valves open, diastolic filling causes vessel wall stretching, increasing vessel volume and pressure. Systole subsequently begins with the rapid contraction of the lymphatic muscle and closure of the upstream valve (Fig. 9). To give readers a clear understanding, we summarize the absorption and transport functions of lymphatic vessels in diverse organs as follows.

##### Lymph absorption and transport in meningeal lymphatic vessels

Although the brain parenchyma is devoid of lymphatic vessels, the rapid clearance of cellular debris and metabolic products in the central nervous system is attributable to the glymphatic system and meningeal lymphatic vessels.^103^ The glymphatic system is composed of three essential components: the para-arterial cerebrospinal fluid influx channel, the para-venous interstitial fluid efflux channel, and the water channel aquaporin-4 in astrocytes that connect these channels.^103^ Cerebrospinal fluid flows into the brain through the para-arterial space and exchanges with interstitial fluid via aquaporin-4; this type of exchange drives metabolite and interstitial fluid into the para-venous space and then into the cerebrospinal fluid circulatory system or directly through the capillary lymphatics into the cervical lymphatics. In particular, the glymphatic system transports lipids within the brain. Excess cholesterol in the brain is eliminated through hydroxylation to 24-hydroxycholesterol by Apolipoprotein E concentrated in astrocytes.

In addition to the glymphatic system, meningeal lymphatic vessels are involved in the elimination of cellular debris and waste products. Dorsal meningeal lymphatic vessels transport macromolecules and cells along the superior sagittal and transverse sinuses. Basal meningeal lymphatic vessels possess lymphatic valves without smooth muscle cells, thereby acquiring a pre-collector phenotype.^104^ Dysfunction of meningeal lymphatic vessels potentially contributes to the onset and progression of Alzheimer’s disease by disrupting the clearance of pathological proteins such as amyloid-β and tau protein, which we will discuss in “Abnormal lymphangiogenesis in human diseases”.

##### Lymph absorption and transport in ocular lymphatic vessels

The ocular surface lymphatic system and Schlemm’s canal regulate lymph absorption and transport in ocular lymphatic vessels. Regarding the ocular surface lymphatic system, lymphatic vessels have been identified in the corneal limbus and conjunctiva of mice and humans.^105^ Ocular surface lymphatic vessels are characterized by button-like junctions, an oak leaf-like shape, and luminal valves with features similar to those of capillary lymphatics and pre-collecting lymphatic vessels.^106^

Schlemm’s canal is an endothelial cell-lined vessel encircling the cornea and abutting the juxtacanalicular region of the trabecular meshwork.^107^ As a special structure regulating intraocular pressure, Schlemm’s canal drains aqueous humor into aqueous and episcleral veins.^108^ Yang et al. described a luminal structure called the lymphatic bridge that connects Schlemm’s canal to ocular surface lymphatic vessels, which allows aqueous humor outflow to the conjunctival lymphatic pathway. This finding suggested, for the first time, that the two lymphatic drainage systems are physically connected, expanding the knowledge of the aqueous humor pathway.^109^

##### Lymph absorption and transport in intestinal lymphatic vessels

Intestinal lymphatic vessels mediate distinct functions in fat absorption, intestinal homeostasis, and peripheral fat transport. Lymphatics continuously deliver nutrients to tissues. Dietary lipids are packaged into chylomicrons in the small intestines and transported via lacteals (capillary lymphatics in the small intestine), where they mix with lymph to become chyle. Mesenteric lymphatic vessels and cisterna chyli carry chyle through the thoracic duct and into the venous circulation.^110^ Defects in lacteals can cause problems with lipid uptake in the intestine. VEGFR2/VEGFR3 signaling, which is mediated through DLL4/NOTCH, is important for lacteal function maintenance. The deletion of DLL4 in lymphatics led to lacteal atrophy and an increase in the number of zipper junctions, resulting in the inability of the tissues to take up chylomicrons.^111^ Interestingly, VEGFA/VEGFR2 signaling also leads to a shift from the establishment of button-like junctions to that of zipper-like junctions in lacteals.^112^ Notably, lacteal atrophy caused by postnatal deletion of VEGFC impaired the absorption of lipids and led to steatorrhea.^113^

Although the small intestine is generally considered an absorptive organ, it can be induced to secrete fluids, causing diarrhea, under certain conditions.^114^ Intestinal lymphatics are important for fluid absorption, especially after a meal. Lymphatics also appear to participate in peripheral tissue lipid balance, and recent studies have shown that they are critical for reverse cholesterol transport. When high-density lipoprotein transports cholesterol out of cells and into peripheral tissues, lymphatic vessels conduct the particles into the bloodstream and back to the liver for excretion through feces.^115^ In mice, obstruction of lymphatic vessels impaired reverse cholesterol transport^116^ and led to increased atherosclerotic plaque formation.^117^

#### Lymphatic vessels in immunity

##### Lymph node lymphatic vessels in coordinating immune responses

Peripheral lymphatic vessels transport antigens and immune cells to draining lymph nodes, fostering an immune response.^100^ In mature lymph nodes, lymphatic vessels and specialized high endothelial venules that are indispensable for the trafficking of naïve lymphocytes into the paracortex of the lymph node,^118^ contributing to the adaptive immune response.^119^ Lymph node LECs contribute to immune response directly by antigen archiving and presentation. Antigen archiving in proliferating lymph node LECs can be directly transferred to other cells, for example, to migratory CD11c^+^ dendritic cells.^120^ Antigens can also be released from dying LECs and subsequently transferred to Batf3-dependent migratory dendritic cells. Antigens can be presented by LECs to immune cells through the MHC (comprising MHCI and MHCII).^121^

**Fig. 10:**
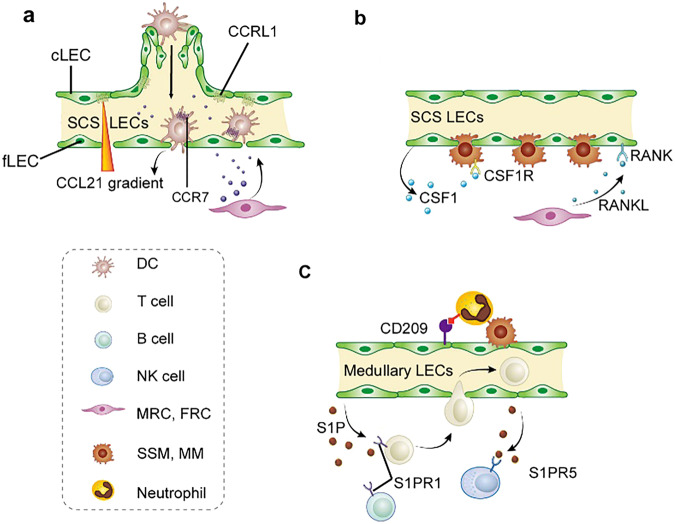
Compartmentalized functions of LN LECs. **a** Decoy CCL21 receptor CCRL1 produced by cLECs creates a CCL21 gradient and regulates intranodal migration of CCR7-expressing DCs; **b** The expression of CSF1 in LECs is maintained by RANK expressed on LECs, which is activated by RANKL produced by MRCs; **c** Medullary LN LECs express CD209 to retain neutrophils, which may be important in clearing lymph-borne pathogens. cLEC ceiling lymphatic endothelial cell, fLEC floor lymphatic endothelial cell, LN lymph node, LEC lymphatic endothelial cell, CCL21 chemokine (C-C motif) ligand 21, CCRL1 chemokine (C-C motif) receptor like 1, CSF1 colony-stimulating factor 1, RANK receptor activator of nuclear factor-kappaB, RANKL receptor activator of nuclear factor-kappaB ligand, DCs dendritic cells, S1P sphingosine 1 phosphate, SCS subcapsular sinus, SSM subcapsular sinus macrophage, MM medullary macrophage, MRC marginal reticular cell. Created with Adobe Illustrator

LECs in different regions can guide immune cell trafficking and positioning within lymph nodes by expressing specific molecules.^122^ For instance, LECs create a chemokine (C-C motif) ligand 21 (CCL21) gradient that facilitates the migration of dendritic cells into the lymph node medulla.^123^ Additionally, S1P gradients, formed by medullary LECs, are necessary for B and T-cell egress into efferent lymphatics and subsequent lymphocyte recirculation. LECs establish a niche for subcapsular sinus and medullary macrophage homing and maintenance. Lymph node LECs serve as major sources of the macrophage pro-survival factor colony-stimulating factor-1, with RANK receptor-expressing LECs inducing colony-stimulating factor-1 expression by interacting with CCL19^+^ marginal reticular cells, producing RANKL (Fig. 10).^124^

##### Lymphatic vessels in central nervous system immunity

Recent studies of meningeal lymphatic vessels revealed an intersection between the immune system and central nervous system.^125^ Meningeal lymphatic vessels may provide a route for central nervous system-derived immune cell and antigen delivery to cervical lymph nodes and thus prevent central nervous system from infection or injury. Meningeal lymphangiogenesis plays an active role in immune surveillance to protect the central nervous system. In the context of glioblastoma, VEGFC-induced meningeal lymphangiogenesis recruited CD8^+^ T cells into the tumor and induced a long-lasting antitumor memory response.^126^ In response to spinal cord injury, VEGFC/VEGFR3 signaling activation-mediated spinal lymphangiogenesis enhanced the immune response by increasing the T cell infiltration rate.^127^ However, studies have shown that meningeal lymphatic vessels potentially promote pathological processes under autoimmune neuroinflammatory conditions. In the multiple sclerosis context, blocking meningeal lymphatic vessels reduced disease severity and alleviated the inflammatory response, likely by interfering with the trafficking and activation of CCR7^+^ T cells in draining lymph nodes.^128^

##### Lymphatic vessels in gut immunity

Intestinal lymphatic vessels are important components of the gut immunosurveillance system, which promotes the mucosal immune response and tolerance. Intestinal dendritic cells present antigens in response to LEC-derived CCL21 production, which is essential for the establishment of oral tolerance.^129^ Intestinal dendritic cells also drive the apoptosis of intestinal epithelial cells in mesenteric lymph nodes by transmitting critical signals that induce Treg cell activation.^129,130^ In addition, different parts of the intestine drain to distinct mesenteric lymph nodes that are immunologically specific to the functional gut segment that they drain. Proximal small intestine-draining mesenteric lymph nodes induce tolerogenic responses, while distal mesenteric lymph nodes induce proinflammatory T cell responses.^131^

The capillary lymphatics in the intestine (also known as lacteals) function as a secondary barrier defending against potential bacterial infections while simultaneously serving as the primary conduit for the dissemination of pathogens, pathogen-derived toxins, and subsequently tissue-derived proinflammatory mediators. DLL4-specific deletion led to lacteal regression and weakened the local immune response, leading to susceptibility to infection and impaired dietary fat uptake.^111^ Additionally, lymphatic drainage can influence the composition of the gut microbiota, with increased drainage potentially promoting a healthier microbiota profile and reducing the incidence of colitis. In contrast, reduced drainage may impede pathogen dissemination and reduce proinflammatory factor levels.

## Abnormal lymphangiogenesis in human diseases

In the above sections, we provided a detailed account of lymphatic vessels in physiological conditions, encompassing lymphatic vessel development and physiological functions. Furthermore, lymphatic vessels actively participate in pathological processes in human diseases. As the comprehension of lymphangiogenesis in diseases improves, the underlying molecular mechanisms have been elucidating. In this section, we provide a comprehensive elucidation of the pivotal roles of lymphangiogenesis in various prevalent diseases and associated regulatory signaling pathways. Meanwhile, lymphangiogenesis in other diseases is concisely presented in Table 3.

**Table 3:** Lymphangiogenesis in other diseases

Disease	Effect of lymphangiogenesis	Molecular mechanisms and regulatory signaling pathways	Reference
Chronic obstructive pulmonary disease	1. Lymphangiogenesis is beneficial in reducing lymphedema and airflow obstruction. 2. Lymphangiogenesis increases immune cell trafficking in patients with advanced chronic obstructive pulmonary disease.	1. Inflammatory cells and epithelial cells promote lymphangiogenesis through the regulation of VEGFC and VEGFD. 2. The expression of CCL21 and chemokine scavenger receptor D6 in LECs of perialveolar lymphatic vessels is increased, which promotes the delivery of immune cells.	^464,465^
Asthma	Impaired lymphangiogenesis disrupts antigen clearance from the lungs and airways.	IL-13 and IL-4 secreted by Th2 cells are identified as potent inhibitors of lymphangiogenesis via JAK/STAT pathways, resulting in the downregulation of PROX1 expression.	^466^
Tuberculosis	1. Lymphangiogenesis induced by Mycobacterium tuberculosis granulomas promotes a systemic T cell response against Mycobacterium tuberculosis antigens. 2. LEC in lymph node provides with the natural niche for Mycobacterium tuberculosis replication.	1. Mycobacterium tuberculosis granulomas promote lymphangiogenesis through the VEGFC/VEGFR3 pathway. 2. Replication of Mycobacterium tuberculosis in the cytoplasmic and phagosome of LECs is dependent on the presence of RD1(encoding ESX-1).	^467,468^
Idiopathic pulmonary fibrosis	1. Perialveolar lymphangiogenesis is positively correlated with the degree of pulmonary fibrosis. 2. Abnormal mural cell coverage of pulmonary lymphatic vessels and impaired lymphatic drainage lead to pulmonary fibrosis.	1. Increased hyaluronic acid and macrophage transdifferentiation promote alveolar lymphangiogenesis. 2. The recruitment of mural cells is facilitated by LECs through the PDGFβ/PDGFRβ pathway, resulting in compromised lymphatic drainage and promoting fibroblast aggregation, ultimately leading to the development of pulmonary fibrosis.	^469,470^
Heart failure	Endogenous cardiac lymphangiogenesis limits cardiac inflammation and perivascular fibrosis, delaying heart faliure development.	Activation of the VEGFC/VEGFR3 signaling prevents the progression to heart failure.	^471,472^
Atherosclerosis	1.Lymphangiogenesis dampens the local inflammatory response at an early stage of plaque development. 2.Disrupted arterial lymphangiogenesis impairs cholesterol efflux from atherosclerotic arteries.	1.Plaque-associated lymphangiogenesis is partly drove by CXCL12/CXCR4 axis. 2.Increased level of RSPO2 binding to LGR4 in atherosclerotic arteries inhibits lymphangiogenesis through impairment of VEGFC-induced AKT/eNOS/NO signaling.	^117,473^
Myocardial infarction	1.Lymphagiogenesis contributes to the fibrosis maturation and scar formation by eliminating excess protein and fluid in granulation and scar phase. 2.Lymphangiogenesis improves cardiac function and suppresses cardiac edema. 3.Lymphoangiocrine signal produced by LECs is cardioprotective, which contributes to reduced cardiomyocytes death and a smaller scarred myocardial area.4.Lymphangiogenesis prompts LEC penetration into the infarcted myocardium, and activated LECs function as intramyocardial immune hubs promote the formation of immunosuppressive microenvironment, facilitating post-myocardial infarction repair.	1.VEGFC expressed by cardiomyocytes induces lymphagiogenesis in/around the infarcted lesion. 2.Epicardial-secreted factor Adrenomedullin drives reparative cardiac lymphangiogenesis and function via CX43. 3.LECs-secreted Reelin regulates cardiomyocytes proliferation and survival through the Integrinβ1 signaling pathway. 4.TBX1 in LECs drives a bi-functional LEC transcriptional program that not only promotes lymphangiogenesis but also enhances the immunosuppressive function which mediated by the expression of CCL21 and ICAM1.	^285,474–476^
Non-alcoholic fatty liver disease	1.Disrupt lymphangiogenesis impedes lymphatic vessel stability and liver homeostasis by regulating fibrosis development and immune cell infiltration. 2. Decreased lymphatic permeability disrupts protein homeostasis and increases inflammation, which is based on impeded LEC metabolism and reorganized cell-cell junctions.	1.OxLDL stimulates the expression of IL-13, thereby inhibiting lymphangiogenesis and affecting lymphatic vessel stability. 2.OxLDL downregulates PROX1, LYVE1, PDPN, and VEGFR3 expression in LECs, which impacts lymphangiogenesis and lymphatic permeability.	^477,478^
Cirrhotic liver	Hepatic lymphangiogenesis promoted by sympathetic nerves prevents liver from portal tract fibrosis.	VEGFC expressed by Schwann cells of sympathetic nerves promotes hepatic lymphangiogenesis.	^479^
Endometriosis	Lymphangiogenesis promotes the infiltration of immune cells, aggravating local chronic inflammation and endometriosis development.	1.IL-1β and TNF-α modulate the overexpression of VEGFC via suppressing COUP-TFII in endometriotic stromal cells, which promotes lymphangiogenesis. 2.Upregulated BST2 regulates the transcription of VEGFC via the activation of NF-κB signaling pathway in endometriotic stromal cells, which promotes lymphangiogenesis.	^480,481^
Arthritis	1.Lymphangiogenesis compensatorily improves joint inflammation during chronic arthritis. 2.Induced lymphangiogenesis improves synovial lymphatic drainage and alleviates arthritis damage in age-related osteoarthritis.	1.Circulating CD11b^+^ myeloid cells infiltrate in joint inflammation, which produce VEGFC to stimulate lymphangiogenesis. 2.Activation of VEGFC/VEGFR3 signaling pathway stimulates synovial lymphangiogenesis.	^482,483^

*VEGF* vascular endothelial growth factor, *VEGFR* vascular endothelial growth factor receptor, *LEC* lymphatic endothelial cell, *IL* interleukin, *CCL* C-C motif chemokine ligand, *Th2* T-helper type 2, *RD1* region of difference 1, *ESX-1* type VII secretion system, *PDGF* platelet-derived growth factor, *CXCL* C-X-C chemokine ligand, *RSPO2* R-spondin 2, *LGR4* leucine-rich repeat-containing G protein-coupled receptor 4, *CX43* connexin 43, *TBX1* T-box 1, *ICAM1* intercellular adhesion molecule 1, *OxLDL* oxidized low-density lipoprotein, *PROX1* prospero homeobox 1, *LYVE1* lymphatic vessel endothelial receptor 1, *PDPN* podoplanin, *TNF* necrosis factor, *COUP-TFII* chicken ovalbumin upstream promoter-transcription factor II, *BST2* bone marrow stromal antigen 2, *NF-κB* nuclear factor-kappa B

### Lymphedema and lymphatic malformation

#### Lymphedema

Lymphedema is a chronic, progressive accumulation of protein-rich fluid in the interstitium due to lymphatic vessel deficiency, resulting in inflammation, fat deposition, and fibrosis. Lymphedema is classified into primary disease caused by congenital abnormalities or secondary disease caused by the injury of iatrogenesis, trauma, and infection. Primary lymphedema, a rare inherited autosomal dominant disorder, is initially characterized by mutations in FLT4 (which encodes VEGFR3). Germline mutations responsible for primary lymphedema have been identified in 28 genes that encode proteins mainly involved in VEGFR3 signaling and these encoded proteins include transcription factors such as GATA2, FOXC2, and SOX18. The majority of mutations result in reduced activation of the VEGFR3 pathway.^110^ Secondary lymphedema arises as a result of infection, trauma or surgery.^132^ Current treatment strategies include mainly physical or surgical interventions to alleviate edema and these treatments include decongestive therapy, intermittent pneumatic compression and liposuction.^133^ In addition, recent studies have revealed the efficacy of pharmacotherapy and cell-based therapies in the treatment of lymphedema.^134,135^

#### Lymphatic malformation

Lymphatic malformation is a benign congenital vascular disease characterized by abnormal lymphatic vessel development. It is caused by single somatic mutations, most of which are in genes encoding components of oncogenic growth factor-activated signal transduction pathways.^136^ The classification of lymphatic malformations is based primarily on clinical manifestations. Cystic lymphatic malformation is the most prevalent type of congenital lymphatic malformation, presenting as solitary lesions of variable sizes. Based on appearance, it is classified into macrocystic, microcystic, or mixed cystic lymphatic malformation.^136^ Most cystic lymphatic malformations are caused by a causative mutation in PIK3CA. Most PIK3CA mutations causing cystic lymphatic malformation activate the phosphatidylinositol-3-kinase (PI3K)/AKT/mTOR signaling cascade.^137^ Complex lymphatic malformations are characterized as multifocal lesions or defection occurring in central collecting lymphatic vessels and show overlapping and variable clinical features. These features include generalized lymphatic anomaly, Gorham-Stout disease, Kaposiform lymphangiomatosis, and central conducting lymphatic anomaly.^138^ Similar to the cause of cystic lymphatic malformation, a causative somatic PIK3CA mutation (His1047Arg) has been reported in generalized lymphatic anomaly.^139^ In other types of complex lymphatic malformations, mutations in genes encoding components of the RAS/MAPK pathway have been reported, including mutations in NRAS and ARAF.^140,141^ Furthermore, germline heterozygous kinase-dead mutations in the gene encoding EPHB4 could activate MAPK signaling in cases of central conducting lymphatic anomaly.^142^ The choice of therapy is based on the location and size of the malformation, and the affected tissues. Pharmacotherapy, surgical resection, sclerotherapy, and thermal ablation can be used to control and relieve symptoms of recurrent effusions, infection, and pain in lymphatic malformations.^136^

### Lymphangiogenesis in cancer

**Fig. 11:**
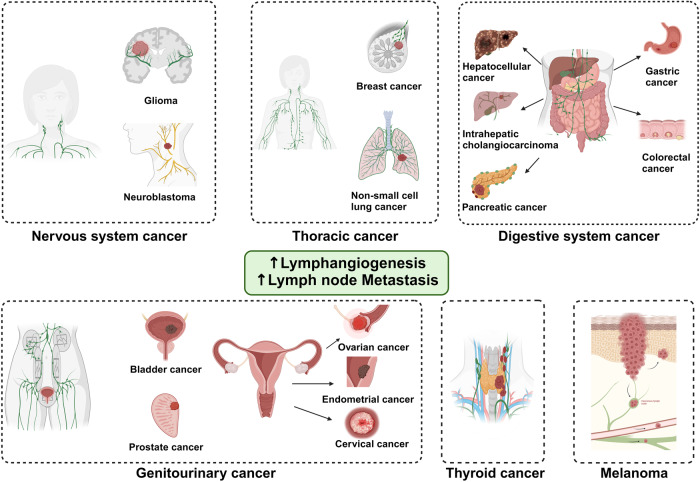
Lymphangiogenesis in cancers. Lymphangiogenesis plays a crucial role in lymph node metastasis, which is associated with poor prognosis and overall survival in a range of malignancies. The molecular mechanisms underlying lymphangiogenesis exhibit diversity across different cancer contexts, potentially suggesting targeted therapeutic strategies for cancers. Created with BioRender.com

**Fig. 12:**
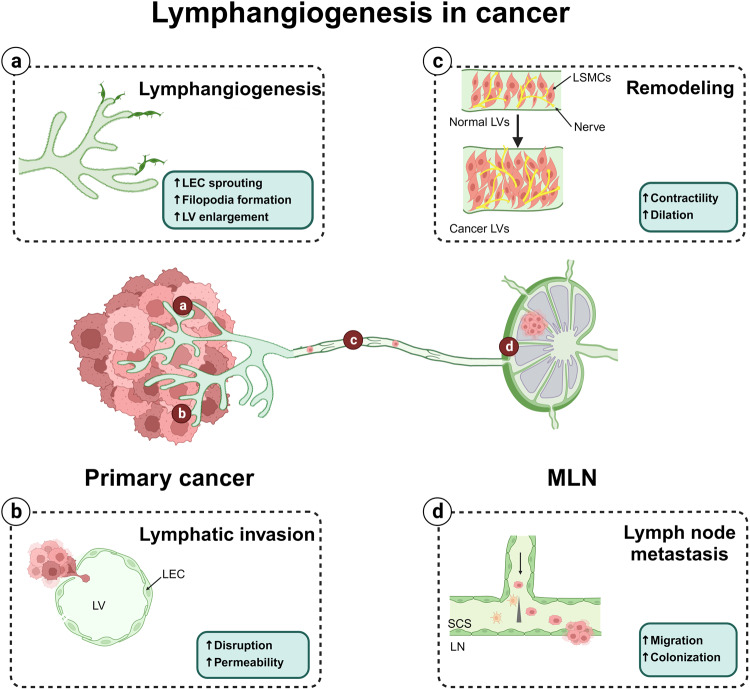
Lymphangiogenesis and lymph node metastasis in cancer. **a** Lymphatic vessels undergo sprouting, filopodia formation, and lymphatic vessel enlargement; **b** The disruption of lymphatic vessels and augmented permeability contribute to the intravasation of cancer cells into lymphatic vessels; **c** An increased coverage by LSMCs and a higher innervation present in the dilated collecting lymphatic vessels, which coordinately enhances collecting lymphatic vessel contractility and pumping frequency; **d** LECs forming the boundaries of the SCS create and maintain chemokine gradients that direct cancer cells to arrive in the SCS of MLN. Furthermore, LECs within the MLN upregulate adhesion molecules, that further support cancer cell colonization. LV lymphatic vessel, LSMC lymphatic smooth muscle cell, LEC lymphatic endothelial cell, SCS subcapsular sinus, MLN metastatic lymph node. Created with BioRender.com

In the past, cancer-associated lymphatic vessels were considered passive transporters of cancer cells. However, recent studies revealed that lymphatic vessels actively participate in the process of cancer metastasis through their dynamic changes, mostly mediated via lymphangiogenesis. Lymphangiogenesis requires the coordination of complex cellular events, including proliferation, sprouting, migration, and tube formation.^46^ Tumor-associated lymphangiogenesis, with its resultant increased permeability and enlargement of lymphatic vessels, synergistically promotes cancer metastasis.^143^ Depending on the type and site of a tumor, the signaling pathways for lymphangiogenesis vary. Moreover, targeting lymphangiogenesis to inhibit cancer metastasis has been proven to be a valuable therapeutic strategy. In this section, we summarize the abnormal lymphangiogenesis in various cancers (Figs. 11, 12).

#### Nervous system cancer

##### Glioma

Malignant glioma is the most common primary cancer of the central nervous system. The expression levels of lymphangiogenic factors (PDPN, VEGFC/D, VEGFR3) are increased in high-grade gliomas, with their expression significantly higher at relapse than it was in the primary tumor.^144^ In addition, microenvironmental stress, including hyperthermia and oxidative stress, has been reported to enhance LEDGF activity, which increases the transcription and expression of VEGFC, thereby promoting lymphangiogenesis.^145^ However, Hu et al. found that meningeal lymphangiogenesis mediated by VEGFC enhanced the efficacy of anti-PD-1/CTLA-4 combination therapy, which was abolished by CCL21/CCR7 blockage.^146^

##### Neuroblastoma

Neuroblastoma is one of the most common cancers in children. Lymphangiogenesis in neuroblastoma is associated with upregulated VEGFC, VEGFD, and VEGFR3, which promotes lymph node metastasis and leads to poor prognosis.^147^ Endogenous soluble VEGFR2 inhibits the activation of VEGFR3 by binding to VEGFC, thereby inhibiting LEC proliferation. In the context of advanced neuroblastoma, the downregulation of sVEGFR2 is correlated with the disease progression.^148^ Furthermore, an anti-VEGFD monoclonal antibody, cVE199, specifically binds VEGFD to inhibit the binding of VEGFD to VEGFR3, thereby significantly reducing the lymphangiogenesis in the context of primary lesions.^149^

#### Thoracic cancer

##### Non-small cell lung cancer

The presence of lymphangiogenesis promoting lymph node metastasis is a crucial determinant for unfavorable prognosis in patients with non-small cell lung cancer. Lymphangiogenesis, indicated by an elevated density of PDPN-positive peritumoral lymphatic vessels, has been demonstrated to be associated with cancer progression and unfavorable prognosis in patients with non-small cell lung cancer.^150^ Watari et al. found that highly metastatic human lung cancer cells have the capability to secrete IL-1, which induces the M2-type polarization of macrophages, augmenting VEGFC expression and subsequently increasing the lymphangiogenesis.^151^ In addition, the overexpression of ITGA6 enhanced the tube formation capacity of LECs, thereby promoting lymphangiogenesis and accelerating subsequent lymph node metastasis in the lung adenocarcinoma context.^152^ Moreover, estrogens in females promoted lymphangiogenesis through an estrogen receptor alpha-dependent pathway in Lewis lung cancer.^153^ Additionally, Hu’s team found that miR-128, functioning as a cancer suppressor, directly targeted VEGFC and subsequently inhibited extracellular signal-regulated kinase (ERK), AKT, and p38 signaling pathway activation to impede lymphangiogenesis in the non-small cell lung cancer context.^154^

##### Breast cancer

The dissemination of breast cancer cells is primarily mediated through lymphatic vessels. Increased lymphatic vessel density has been associated with lymph node metastasis and poorer survival in patients with breast cancer.^155^ The interaction between PDPN and LEC-derived galectin-8 contributed to the activation of promigratory integrin β1, thereby augmenting PDPN-expressing macrophages proximal to lymphatics, which subsequently stimulated local matrix remodeling and promoted lymphangiogenesis.^156^ Weichand et al. revealed that S1PR1 on tumor-associated macrophages promoted lymphangiogenesis and metastasis via NLRP3/IL-1β.^157^ In addition, Zheng et al. found that the hypomethylation of the long noncoding RNA (lncRNA) HUMT promoted lymphangiogenesis and metastasis by enhancing FOXK1 transcription to activated the AKT/mTOR and VEGFC signaling pathways in breast cancer cells.^158^ A recent study conducted by Li et al. revealed that the transcription factor ZKSCAN5 directly interacted with SETD7, forming a complex at the VEGFC promoter. This interaction effectively regulated the expression of VEGFC, inducing tube formation of LECs and promoting lymphangiogenesis.^159^ Furthermore, NADPH oxidase 4 promoted lymphangiogenesis via the ROS/ERK/CCL21 signaling pathway and providing the entry for metastasis of CCR7-expressing breast cancer cells.^160^ Nectin-4 induced chemotactic interactions between CXCR4-expressing cancer cells and CXCL12-expressing LECs, which stimulated VEGFC and LYVE1 expression to promote LEC proliferation and migration, ultimately promoting lymphangiogenesis.^161^ Additionally, heat shock protein 90α significantly enhanced the migration and tube formation abilities of LECs to promote lymphangiogenesis via the LRP1/AKT/CXCL8 signaling pathway.^162^ Chemotherapy remains an effective treatment for breast cancer, although its efficacy may be influenced by lymphangiogenesis. Harris and colleagues found that lymphangiogenesis induced by platinum chemotherapy increased the rate of lymph node metastasis in breast cancer, which was prevented by adjuvant anti-VEGFR3 therapy.^163^

#### Digestive system cancer

##### Hepatocellular cancer

Hepatocellular cancer is the most common type of liver cancer.^164^ High lymphatic vessel density has been associated with reduced survival and disease-free survival of patients with hepatocellular cancer.^165^ VEGFD-overexpressing hepatocellular carcinoma cells promote lymphangiogenesis, resulting in an increased rate of lymph node metastasis.^166^ Furthermore, lncRNA HANR, which regulates exosomal miR-296 secretion, may induce lymphangiogenesis via the EAG1/VEGFA axis in LECs.^167^ Targeting lymphangiogenesis has been proposed as a promising approach for suppressing hepatocellular cancer metastasis. An IgG-like fusion protein molecule (VEGF receptor 31-immunoglobulin, VEGFR31-Ig) binding VEGFC has been developed to inhibit lymphangiogenesis, thereby suppressing cancer growth and metastasis.^168^

##### Intrahepatic cholangiocarcinoma

Intrahepatic cholangiocarcinoma is an aggressive and lethal type of primary liver cancer.^169^ Lymphangiogenesis has been correlated with an increased risk of lymph node metastasis and reduced overall survival in patients with intrahepatic cholangiocarcinoma.^170^ Carpino et al. found that THB1, THBS2, and PEDF released into the intrahepatic cholangiocarcinoma stroma induced lymphangiogenesis, which contributed to preferential lymph node metastasis of intrahepatic cholangiocarcinoma.^171^ Furthermore, cholangiocarcinoma-derived PDGFD activated LEC-adjacent fibroblasts, which secreted VEGFC and VEGFA, resulting in lymphangiogenesis to promote cancer cell intravasation.^172^ Moreover, PDGF-BB secreted by cancer-associated fibroblasts activated the receptor PDGFR-β and downstream ERK1/2-JNK signaling pathways in LECs to promote lymphangiogenesis.^173^

##### Pancreatic cancer

The high lethality of pancreatic cancer stems from its propensity for rapid dissemination in the lymphatic system and distant organs, and lymph node metastasis may occur early in the course of pancreatic cancer development.^174^ Similar to its effect on other cancer types, VEGFC may mediate lymphangiogenesis in the context of pancreatic cancer. Under hypoxic conditions, BANCR is overexpressed in pancreatic cancer and promotes lymphangiogenesis by upregulating the HIF-1α/VEGFC/VEGFR3 pathway.^175^ In contrast, circular RNA (circRNA) circNFIB1 exerts a suppressive effect on lymphangiogenesis by downregulating the effect of miR-486-5p and upregulating PIK3R1 expression to inhibit VEGFC signaling.^176^ VEGFC could be secreted from cells in extracellular vesicles to facilitate lymphangiogenesis. Wang et al. reported that the downregulation of DUSP2 in pancreatic cancer enhanced extracellular vesicle-associated secretion of VEGFC. Thus, VEGFC enhances lymphangiogenesis and cancer cell invasion into lymphatic vessels through paracrine and autocrine mechanisms, ultimately leading to lymph node metastasis.^177^ Additionally, based on the aforementioned mechanism, Wang et al. reported a selective inhibitor B390 of HDAC1/2 that effectively suppressed lymphangiogenesis by reinstating DUSP2 expression.^178^ Other studies have been aimed at understanding lymphangiogenesis independent of VEGFC action. KRAS(G12D)-mutated pancreatic cancer cells maintained the secretion of extracellular vesicles carrying hnRNPA1 in a SUMOylation-dependent manner, thereby promoting lymphangiogenesis through the stabilization of PROX1 mRNA in vesicle-recipient LECs.^179^ Furthermore, Zhou et al. demonstrated that exosomes derived from pancreatic cancer cells exerted a stimulating effect on the proliferation and migration of LECs via the downregulating ABHD11-AS1 expression, to promote lymphangiogenesis.^180^ With the recent development of the “medicine-industry combination”, Shen et al. utilized three-dimensional imaging technology to observe the relationship between primary pancreatic cancer and lymphatic vessel networks, revealing peritumoral lymphangiogenesis, laying a technological and morphological foundation for future systematic detection and three-dimensional analysis of lymphatic invasion in tumor microenvironment (TME).^181^

##### Gastric cancer

Lymph node metastasis represents the predominant pattern of gastric cancer dissemination, with lymphangiogenesis emerging as a pivotal contributing factor. The evidence suggests that gastric cancer cells stimulate lymphangiogenesis through the secretion of VEGFC, a process typically facilitated by other regulatory factors, such as transforming growth factor β1 (TGFβ1) and MACC1, in the TME.^182,183^ Our recent study reported that CRIP1 reshaped the gastric TME to increase the lymphangiogenesis and lymphatic vessel permeability by increasing the amount of secreted VEGFC and CCL5.^184^ Additionally, tumor-associated lymphangiogenesis and lymph node metastasis is influenced by metabolic factors. Yang et al. demonstrated that the elevation of oxLDL levels in plasma induced the activation of the nuclear factor (NF)-κB pathway through binding with LOX-1, leading to the upregulation of VEGFC expression and subsequently facilitating lymphangiogenesis.^185^ The rate-limiting enzyme SOAT1 in the cholesterol metabolic pathway exerts effect by upregulating the SREBP1 and SREBP2 expression.^186^ Furthermore, recent studies have also reported several factors that impede lymphangiogenesis in gastric cancer. Among them, kallistatin exerts its inhibitory effects on lymphangiogenesis and lymphatic node metastasis by downregulating the expression and secretion of VEGFC through the LRP6/IKK/IκB/NF-κB pathway.^187^ Furthermore, some compounds, such as auramycin G and Babao Dan, exert inhibitory effects on lymphangiogenesis through the downregulation of VEGFC, and curcumin hinders lymphangiogenesis by targeting the HMGB1/VEGFD axis.^188–190^

##### Colorectal cancer

Colorectal cancer, a prevalent gastrointestinal malignancy, frequently progresses to lymph node metastasis. Lymphangiogenesis indicates a higher risk of local recurrence and poorer prognosis in patients with colorectal cancer.^191^ Metastasis-associated protein 1 has been demonstrated to induce VEGFC expression and promote lymphangiogenesis in colorectal cancer.^192^ Moreover, Xiang’s team found that colorectal cancer-derived exosomes promoted lymphangiogenesis in sentinel lymph nodes through IRF-2, which induced VEGFC expression in macrophages.^193^ CCBE1, expressed and secreted by colorectal cancer cells and cancer-associated fibroblasts, facilitated lymphangiogenesis through the promotion of VEGFC proteolysis and maturation, while its activity was negatively regulated by TGFβ signaling.^194^ The development of therapeutic approaches targeting lymphangiogenesis for the treatment of colorectal cancer is currently ongoing. Qingjie Fuzheng Granule and Pien Tze Huang exhibit inhibitory effects on tumor growth and lymphangiogenesis via the PI3K/AKT/VEGFC pathway.^195,196^ In addition, an ethanol extract of Hedyotis diffusa Willd suppresses VEGFC-stimulated LEC migration and tube formation while concurrently downregulating the expression of downstream molecules involved in the VEGFC/VEGFR3 signaling pathway, such as MMP2, MMP9, cyclin D1, and CDK4.^197^

#### Genitourinary cancer

##### Bladder cancer

Bladder cancer is a highly prevalent malignancy in the genitourinary system.^164^ Higher lymph vessel density has been correlated with decreased metastasis-free survival of patients with bladder cancer.^198^ Changhao Chen and colleagues found that the lncRNA LINC01296, termed LNMAT1, epigenetically induced CCL2 expression in bladder cancer cells, recruiting macrophages to the TME, which induced lymphangiogenesis via VEGFC secretion.^199^ Afterward, the team identified another lncRNA known as LNMAT2 in exosomes secreted by bladder cancer cells that stimulated LEC tube formation and migration via epigenetically acting on the PROX1 promoter to upregulate the expression.^200^ Subsequently, Chen et al. discovered that EV-mediated ELNAT1 also promoted lymphangiogenesis via the transcriptional upregulation of SOX18 expression in human LECs via SUMOylation motification.^201^ In addition, overexpression of the lncRNA BLACAT2 stimulated lymphangiogenesis by binding WDR5, the core subunit of human H3K4 methyltransferase complexes, to increase the expression of VEGFC.^202^ The novel circRNA circEHBP1 mediated TGFβR1 overexpression and activated the TGFβ/SMAD3 signaling pathway in bladder cancer cells, thereby promoting the secretion of VEGFD to drive lymphangiogenesis.^203^ The inhibition of lymphangiogenesis is a promising therapeutic strategy for impeding lymph node metastasis in patients with bladder cancer. Blocking the VEGFC/D signaling pathways by administering soluble VEGFR3 encoded by adenovirus or using clodronate liposomes for depletion of tumor-associated macrophages may markedly inhibit lymphangiogenesis in patients with bladder cancer.^204^

##### Prostate cancer

The incidence of prostate cancer is highest among all the malignancies affecting the male reproductive system. Previous studies have demonstrated that elevated lymphatic vessel density in conjunction with increased VEGFC expression was indicative of metastasis and unfavorable prognosis in patients diagnosed with prostate cancer.^205^ Lysophosphatidic acid increased the expression of VEGFC and promoted lymphangiogenesis by upregulating calreticulin in prostate cancer cells.^206^ Relevant studies are currently ongoing to investigate the potential of targeted lymphangiogenesis therapy in managing lymph node metastasis and distant metastasis of prostate cancer. The administration of a VEGFC ligand trap (soluble VEGFR3) or anti-VEGFR3 antibody (mF4-31C1) resulted in a significant reduction in the lymphangiogenesis, as well as metastasis to regional lymph nodes and distant organs.^207^ Yano et al. revealed that glucocorticoids suppressed lymphangiogenesis by downregulating the expression of VEGFC.^208^

##### Ovarian cancer

Ovarian cancer is one of the most aggressive gynecological cancers and is associated with poor prognosis. High lymphatic vessel density has been found to be significantly associated with lymph node metastasis and recurrence in patients with ovarian cancer.^209^ Sapoznik et al. found that follicle-stimulating hormone enhanced the interaction between LEDGF and the VEGFC promoter, thereby increasing VEGFC expression and promoting lymphangiogenesis.^210^ Additionally, the overexpression of Her-2/NEU increased the endothelial permeability and facilitated lymph node metastasis by upregulating the expression of VEGFC.^211^ Cheng and his colleagues found that hypoxia-induced secretion of HIF-1α facilitated lymphangiogenesis and expedited metastasis through the ALKBH5/m6A-ITGB1/FAK signaling pathway.^212^ Other factors modulate lymphangiogenesis to influence the rate of lymph node metastasis in ovarian cancer. For example, SPARC, a Ca^2+^-binding matricellular glycoprotein, has been demonstrated to inhibit lymphangiogenesis by downregulating VEGFC and VEGFD expression.^213^

##### Cervical cancer

Patients with cervical cancer and lymph node metastasis face a dismal prognosis, and lymphangiogenesis promotes the dissemination of cervical cancer cells to regional lymph nodes. Chen et al. found that TNFα promoted lymphangiogenesis by VEGFC-mediated activation of the AKT and ERK pathways, which was suppressed by MAZ51, a VEGFR3 inhibitor.^214^ PTPRM, which has been identified as an independent prognostic factor of patients with cervical cancer, can induce VEGFC-dependent lymphangiogenesis.^215^ Upregulated expression of FASN and FABP5, which both play important roles in lipid metabolism, has been positively correlated with the lymph node metastasis of cervical cancer.^216,217^ As a pivotal enzyme in lipid metabolism, FASN stimulated the secretion of PDGF-AA and IGFBP3 by cervical cancer cells, thereby facilitating lymphangiogenesis and promoting metastasis.^216^ Furthermore, the upregulation of FABP5 reprogrammed fatty acid metabolism, leading to an increase in intracellular fatty acids that activated the NF-κB pathway, resulting in lymphangiogenesis in cervical cancer.^217^ Additionally, exosomal miR-221-3p secreted by cervical cancer cells was transferred to LECs, thereby activating the ERK and AKT pathways through downregulation of VASH1 and promoting lymphangiogenesis.^218^ A recent study reported that circVPRBP interacted with RACK1 and shielded the S122 O-GlcNAcylation site, thereby inducing RACK1 degradation, which subsequently suppressed galectin 1-mediated lymphangiogenesis and lymph node metastasis in cervical cancer.^219^

#### Papillary thyroid cancer

Lymphangiogenesis has been demonstrated to facilitate lymph node metastasis in papillary thyroid cancer. Choi et al. found that increased lymphatic vessel density, as assessed by high PDPN and VEGFR3 expression, was significantly associated with the incidence of lymph node metastasis in the papillary thyroid cancer context.^220^ Additionally, recurrent papillary thyroid cancer exhibited higher peritumoral lymphatic vessel density than nonrecurrent thyroid cancer.^221^ Moreover, lncRNA MFSD4A-AS1 functioned as a competing endogenous RNA to disrupt miRNA-mediated VEGFA/C repression and activated TGFβ signaling, thereby promoting lymphangiogenesis.^222^

#### Melanoma

Melanoma is an extremely malignant cutaneous cancer with a high propensity for metastasis, predominantly through lymph node metastasis. The prognostic significance of lymphatic vessel density in melanoma has been documented, with higher densities associated with a poorer prognosis.^223^ Melanoma-derived melanosomes facilitate the transfer of let-7i to LECs, thereby triggering type I IFN signaling and promoting lymphangiogenesis.^224^ Moreover, melanoma cells secrete extracellular vesicles enriched with NGFR, which promotes lymphangiogenesis and facilitate cancer cell adhesion through the induction of ERK kinase activity, activation of NF-κB, and upregulation of ICAM-1 expression in LECs.^225^ The high expression of CD147 within melanoma cells has been reported to stimulate lymphangiogenesis through the upregulation of PROX1 expression.^226^ Additionally, both Adrenomedullin and Apelin have been demonstrated to enhance lymphangiogenesis in melanoma.^227,228^ In the context of melanoma, claudin-3 exerted an inhibitory effect on lymphangiogenesis through the downregulation of VEGFC and PI3K signaling pathways.^229^ Additionally, various inhibitors targeting lymphangiogenesis have been documented, including rapamycin and a novel 2-aminobenzimidazole derivative called MFB, which effectively suppressed lymphangiogenesis by downregulating VEGFs.^230,231^ Interestingly, Sasso et al. demonstrated that the induction of lymphangiogenesis through VEGFC increased the efficacy of immunotherapy, thereby presenting a novel therapeutic strategy for cancer treatment targeting lymphangiogenesis.^232^

### Alzheimer’s disease

Alzheimer’s disease is the most common form of dementia, in which impaired amyloid-β clearance from the brain is the core etiology.^233^ Meningeal lymphatics are involved in the clearance of molecules, including amyloid-β (as explained in “Lymph absorption and transport in meningeal lymphatic vessels”), and their dysfunction is an aggravating factor in Alzheimer’s disease pathology.^234^ Modulation of lymphatic vessel function might be a novel therapeutic strategy for Alzheimer’s disease. Recent studies have shown that VEGFC treatment promoted meningeal lymphangiogenesis in transgenic mice, which decreased the level of soluble amyloid-β in cerebrospinal fluid.^234,235^ Moreover, the therapeutic delivery of VEGFC enhanced meningeal lymphatic function to increase the clearance rate of amyloid-β by monoclonal antibodies.^236^

### Ocular hypertension and glaucoma

Glaucoma is the foremost cause of irreversible blindness, with elevated intraocular pressure being the most crucial risk factor. Ocular lymphatic vessels and Schlemm’s canal play pivotal roles in regulating intraocular pressure under the physiological condition (as outlined in “Lymph absorption and transport in ocular lymphatic vessels”). VEGFC/VEGFR3 plays a crucial role in the development and maintenance of Schlemm’s canal. The application of recombinant VEGFC resulted in the promotion of Schlemm’s canal growth in mice and led to a tendency toward decreased intraocular pressure.^237^ Kim et al. found that Schlemm’s canal integrity was maintained via the interconnected and coordinated functions of ANG/TIE2 signaling, aqueous humor outflow and PROX1 activity. Deletion of ANG1/ANG2 or TIE2 severely impaired the integrity of Schlemm’s canal, resulting in elevated intraocular pressure, retinal neuron damage, and impaired retinal ganglion cell function, all hallmarks of primary open-angle glaucoma.^238^ Recently, SVEP1 was identified as a modifier of TEK expression during Schlemm’s canal development and affected the penetrance and severity of primary congenital glaucoma disease.^239^ However, the underlying mechanism through which ocular lymphatic dysfunction contributes to the pathogenesis of glaucoma remains unclear, and further investigations are needed to gain a comprehensive understanding.

### Obesity and lipedema

Obesity is characterized by excessive adipose tissue accumulation resulting from an imbalance between energy intake and expenditure.^240^ Adipose tissue is no longer considered solely an energy storage depot but has been recognized as an active endocrine organ that secretes numerous adipokines and proinflammatory cytokines.^241^ Obesity is widely acknowledged to be a significant clinical risk factor for the development of lymphedema. Obesity-induced chronic inflammation, fibrosis, and increased adipose tissue deposition detrimentally impact lymphatic vessel function, thereby exacerbating the inflammatory response and precipitating the development of lymphedema.^242^ A decrease in lymphatic vessel density has also been observed in murine models of obesity.^243^ Therefore, maintaining a normal number and function of lymphatic vessels may potentially ameliorate the metabolic disruption in obese patients. The study conducted by Chakraborty et al. substantiated this possibility, demonstrating that an increased VEGFD-induced lymphangiogenesis in adipose tissue mitigated the obesity-related immune accumulation and increased the metabolism.^244^ Subsequently, they observed that VEGFD-induced lymphangiogenesis in adipose tissue resulted in a decrease in the macrophage populations and accelerated systemic fatty acid utilization, thereby facilitating the remodeling of the inflammatory response.^245^

Lipedema, often misidentified as lymphedema or obesity, is a chronic progressive disease characterized by disproportional adipose tissue distribution and limb pain, predominantly in women. Patients with lipedema present with features of lymphedema, particularly in advanced stages, which may arise from the synergistic effects of lymphatic abnormalities and lipid accumulation. Amann et al. employed fluorescence microlymphography and thus identified beadlike dilated lymphatic vessels in individuals with lipedema.^246^ In addition, Lohrmann et al. revealed functional and morphological aberrations in the lymphatic vessels of lower extremities in lipedema patients.^247^ In terms of lymphatic abnormalities as potential etiologies for lipedema, the leakage of lymph fluid has been shown to promote adipocyte proliferation significantly.^248^ However, imbalanced and prolonged adipose tissue expansion may contribute to abnormal contraction of collecting lymphatic vessels, as observed by Blum et al. in mice chronically fed a high-fat diet.^249^ Expanded adipocytes secreted certain lymphangiogenic factors, such as VEGFC, VEGFD and ANG2.^250^ In light of these findings, further investigations are warranted to elucidate the intricate association between lipedema and lymphatic vessel impairment.

### Diabetes mellitus

Diabetes mellitus is a chronic metabolic disorder characterized by hyperglycemia and abnormal carbohydrate, fat, and protein metabolism.^251^ Impaired lymphangiogenesis is a complication of diabetes mellitus. Wenstedt et al. observed that salt-sensitive increases in blood pressure in individuals with Type 1 diabetes may be linked to the absence of macrophages and thus reduced lymphangiogenesis.^252^ Wu et al. demonstrated that diabetes-induced ROS-activated c-Src-dependent phosphorylation of VEGFR3 and upregulation of epsin expression, causing VEGFR3 degradation via the interaction between epsin and VEGFR3, ultimately leading to impaired lymphangiogenesis.^253^ In patients with Type 2 diabetes, increased lymphatic permeability has been demonstrated to lead to lymphatic vessel dysfunction. Scallan et al. initially identified increased permeability in the collecting lymphatic vessels in individuals with Type 2 diabetes, leading to lymphatic leakage. This outcome was attributed to the diminished bioavailability of NO, which was rectified by inhibiting the action of the NO-degrading enzyme phosphodiesterase 3.^254^ Similarly, Cifarelli et al. observed that the downregulation of VEGFC/VEGFR2/AKT after CD36 silencing resulted in VE cadherin degradation and subsequently increased lymphatic vessel permeability in individuals diagnosed with Type 2 diabetes.^255^ The prolonged hyperglycemia associated with diabetes can cause oxidative stress, advanced glycation end-product formation, and inflammation, which can lead to significant damage to various organs, such as the kidneys and eyes.^256^ Recent studies have demonstrated the involvement of lymphatic vessels in the pathogenesis of these complications.

#### Diabetic kidney disease

Hyperglycemia and other metabolic abnormalities in diabetes can lead to ultrastructural and functional changes in the glomeruli and tubules, eventually causing diabetic kidney disease.^257^ A study by Kim et al. showed that inhibiting lymphangiogenesis alleviated lipid overload induced by diabetic kidney disease and relieved symptoms.^258^ Mechanistically, this outcome was caused by lymphangiogenesis in diabetic kidney disease, which promoted tubulointerstitial fibrosis in the kidneys.^259^ Subsequently, Hwang et al. demonstrated that the mitigation of kidney damage in diabetic kidney disease was achievable through targeted inhibition of lymphangiogenesis using a specific VEGFR3 inhibitor.^260^ Collectively, these studies indicated that targeting lymphangiogenesis-related factors may be a viable therapeutic strategy for diabetic kidney disease.

#### Diabetic retinopathy

Diabetic retinopathy is a common ocular complication of diabetes that can eventually lead to vision loss.^261^ Aberrant lymphangiogenesis has been observed to be involved in the pathogenesis of diabetic retinopathy.^262,263^ Gucciardo et al. observed that the microenvironment of diabetic retinopathy can promote pathological lymphangiogenesis.^264^ Korhonen et al. identified significant enrichment of genes and signaling pathways associated with lymphatic vessel development in tissues obtained from patients diagnosed with diabetic retinopathy through mRNA sequencing and Gene Ontology and pathway enrichment analyses.^265^ However, the mechanisms underlying pathological lymphangiogenesis contributions to the pathogenesis of diabetic retinopathy are currently unknown, and further investigation is required.

#### Diabetic wound healing

Lymphatic dysfunction in diabetes mellitus may lead to the impaired transport of immune cells, growth factors, and other molecules involved in tissue regeneration, resulting in delayed wound healing.^266^ Several studies have shown that the activation of lymphangiogenesis facilitates diabetic wound healing.^267,268^ Topical simvastatin or negative-pressure wound therapy are potential therapeutic approaches to promote wound healing in people with diabetes mellitus, and the mechanisms underlying these therapies both involve the promotion of lymphangiogenesis.^269–271^ In addition, some lncRNAs have also been shown to promote diabetic wound healing by promoting lymphangiogenesis.^272,273^ Considering these findings, researchers engineered a new tissue material to induce lymphangiogenesis, paving the way for the development of novel strategies to facilitate diabetic wound healing.^274^

### Wound healing

Wound healing is a complex and dynamic process that involves a series of overlapping stages to restore tissue integrity and function. It encompasses revascularization, inflammation, innervation, and remodeling.^275^ Activation of lymphangiogenesis pathways is considered an effective strategy to facilitate healing of chronic wounds and alleviate tissue inflammation, which is mainly induced by VEGFC/VEGFR3 signaling.^276^ During the wound healing process, platelets promote the release of VEGFC, thereby facilitating lymphangiogenesis. Immune cells, proteins, and fluids are transported out of the wound area through newly formed lymphatic vessels.^277^ Stabilized expression of HIF-1α in a wound also promotes the expression of VEGFC, thereby regulating lymphangiogenesis.^278^ Hosono et al. found increased expression of COX-2 and mPGES-1 at wound sites and showed that these proteins promoted the expression of VEGFC and induced lymphangiogenesis.^279^ Notably, impaired or insufficient lymphangiogenesis may result in hindered or incomplete wound healing.^280^

In addition, lymphatic vessels form a specific niche for the regeneration of diverse tissues. Dermal capillary lymphatic vessels regulate the regeneration of hair follicles by dynamically interacting with stem cells. Mechanically, activated hair follicle stem cells express ANG-like protein 4 to promote capillary lymphatic vessel remodeling and reduce lymph drainage to initiate hair follicle regeneration.^281,282^ Skeletal lymphatic vessels mediate the regeneration of bone and hematopoietic stem cells under genotoxic stress conditions. Biswas et al. recently found that skeletal lymphangiogenesis specifically recruited and enhanced the proliferation of mature Myh11^+^CXCR4^+^ pericytes via secreting CXCL12, inducing their differentiation into osteoblasts and contributing to bone regeneration.^283^ Moreover, they found through experiments that inhibition of skeletal lymphangiogenesis reduced the proportion of hematopoietic stem cells, indicating a particularly potent regenerative function of lymphatic vessels.^283^ Additionally, lymphatic vessels have been shown to be indispensable for heart regeneration after myocardial infarction. Gancz et al. found that the lack of cardiac lymphatic vessels impaired heart regeneration by preventing VEGFC/VEGFR3 signaling in zebrafish.^284^ Epicardium-derived Adrenomedullin signaling in mice stimulated regenerative cardiac lymphangiogenesis via lateralization of CX43, which is a potential therapeutic target for cardiac regeneration.^285^

### Transplant rejection

Types of transplant rejection include host-versus-graft disease (HVGD) and graft-versus-host disease (GVHD). HVGD is an immune-mediated response wherein the recipient’s immune system discerns the transplanted organ as an exogenous entity and subsequently initiates an immunological assault against it.^286^ Lymphatic vessels play crucial roles as conduits for antigen-presenting cells and soluble antigens, thereby facilitating their transport. Surgical intervention resulting in disrupted lymphatic vessel integrity can lead to impaired lymphatic drainage, consequently impacting both acute and chronic rejection responses after transplantation. Moreover, lymphangiogenesis exhibits heterogeneity in transplant rejection among organs.^287^ During ischemia-reperfusion injury in rat heart allografts, activation of the VEGFC/VEGFR3 axis induced lymphangiogenesis and subsequently exacerbated allograft inflammation.^288^ After the transplantation of minor antigen sex-mismatched murine heart grafts, an increase in the lymphatic flow index was associated with higher lymphatic vessel density and inflammatory infiltration of T cells and macrophages.^289^ VEGFR3 is involved in the trafficking of immune cells from peripheral tissues to secondary lymphoid organs by regulating the production of CCL21 in allogeneic lymphatic vessels. Adenovirus VEGFR3-Ig inhibited lymphangiogenesis and attenuated cardiac allograft rejection by reducing the number of transported and activated antigen-presenting cells.^290^ Additionally, Kerjaschki et al. demonstrated that lymphangiogenesis contributed to nodular mononuclear infiltration while also played a role in sustaining a potentially detrimental alloreactive immune response in hosts after renal transplant.^291^ Mechanistically, the nodular infiltrates contained a significant number of CCR7-positive immune cells, which appeared to be attracted by SLC/CCL21 produced and released by LECs.^291^ However, it has been suggested that lymphangiogenesis plays a pivotal role in enhancing the survival of allografts. Hyaluronic acid has been identified to induce inflammation and contribute to the development of chronic allograft rejection.^292^ Cui et al. found that stimulation of lymphangiogenesis using VEGF-C156S, a mutant form of VEGFC selectively binding to VEGFR3, resulted in the attenuation of an established rejection response and increased the clearance of hyaluronic acid from lung allografts.^293^ Pedersen et al. found that lymphangiogenesis in a mouse model of renal transplant rejection extended the lifespan of the recipients, which may have been related to immune tolerance promoted by lymphangiogenesis.^294^

GVHD is an immune response of immunocompetent cells in the graft against histocompatibility antigens in the host, resulting in damage to the host. Acute GVHD is triggered by alloreactive T cells that damage peripheral tissues and lymphoid organs.^295^ Gehlsen et al. found that lymphangiogenesis is involved in the pathogenesis of ocular GVHD.^296^ In addition, Mertlitz et al. found that acute GVHD was associated with lymphangiogenesis in murine allo-HSCT models and in intestinal tissue biopsy samples taken from patients, while the administration of anti-VEGFR3 antibodies suppressing lymphangiogenesis ameliorated GVHD and prolonged the survival in murine models.^297^

## Therapeutic interventions and clinical research progress of lymphangiogenesis

Many past and present efforts have been made to study and reveal the interventions of lymphangiogenesis under physiological and pathological conditions. In the following section, we summarize the known promoters and inhibitors of lymphangiogenesis, list therapeutic interventions, and describe the progress in their clinical applications.

### Interventions of lymphangiogenesis

#### VEGFs

##### Agonistic effects of VEGFs

**Fig. 13:**
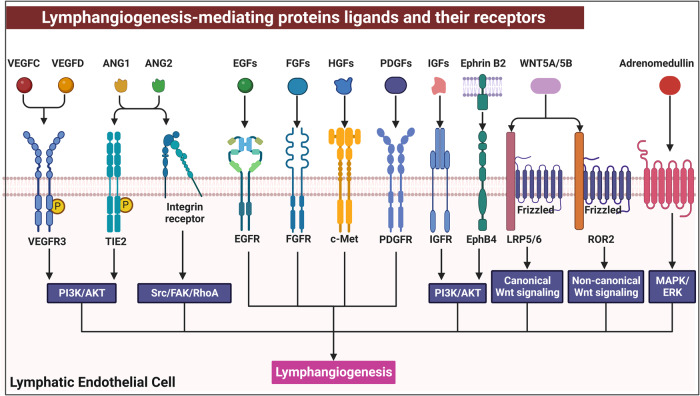
Lymphangiogenesis-mediating proteins ligands and their receptors. Schematic diagram showing the main promoters of lymphangiogenesis with soluble ligands or interacting proteins present outside the cell and the transmembrane receptors expressed by lymphatic endothelial cells (LECs) at 7the cell surface. VEGFC vascular endothelial growth factor C, VEGFD vascular endothelial growth factor D, VEGFR3 vascular endothelial growth factor receptors 3, ANG angiopoietin, TIE, tunica interna endothelial cell kinase, EGF epidermal growth factor, EGFR epidermal growth factor receptor, FGF fibroblast growth factor, FGFR fibroblast growth factor receptor, HGF hepatocyte growth factor, PDGF platelet-derived growth factor, PDGFR platelet-derived growth factor receptor, IGF insulin-like growth factor, IGFR insulin-like growth factor receptor, AM adrenomedullin. Created with BioRender.com

Among VEGF family members, VEGFC and VEGFD are the best-characterized and specific growth factors for lymphatic vessels. Generally, VEGFC and VEGFD function as ligands that bind receptors on the LEC membrane and then activate downstream signaling cascades to promote lymphangiogenesis. VEGFC and VEGFD both bind to the receptor VEGFR3, which is commonly expressed by LECs.^298,299^ After binding of VEGFC or VEGFD, VEGFR3 dimerizes and is phosphorylated, leading to the activation of its cytoplasmic tail tyrosine kinase activity. The phosphorylation of VEGFR3 leads to the recruitment of some important proteins, such as GRB2, CRK, and SHC, which mediate the activation of downstream signaling pathways, including the conserved PI3K/AKT, MAPK/ERK, and MAPK/JNK pathways, etc.^300,301^ Activation of these molecular signaling cascades promotes the initiation of a series of cellular biological behaviors, such as LEC proliferation and migration and vessel sprouting (Fig. 13). VEGFA has been identified as the angiogenic factor acting via VEGFR1 and VEGFR2. Some recent work revealed that VEGFA promotes LEC proliferation and migration and lympangiogenesis.^302–305^ On one hand, VEGFA could function on VEGFR1/2 expressed by LECs and promote lymphanigongenesis.^304,305^ On the other hand, VEGFA could indirectly induce lymphangiogenesis via recruitment of VEGFR1^+^ bone marrow-derived macrophages which in turn release both hemangiogenic and lymphangiogenic growth factors.^302,303^

##### Targeted inhibitors of VEGF signaling

Antibody drugs: Bevacizumab is a well-characterized monoclonal antibody of VEGFs and is used as an anti-angiogenic drug for various types of cancer. Moreover, some clinical trials have been carried out to evaluate its anti-lymphangiogenic effects.^306^ Dumond et al. reported a new VEGFC antibody named 1E9 that significantly inhibited VEGFR3 signaling activation in LECs.^307^ Similarly, VGX-100, another highly specific monoclonal antibody, targeted VEGFC and impaired VEGFC-VEGFR2/3 signaling activation.^308^ A soluble fusion protein VEGFR3-immunoglobulin that bound VEGFC and inhibited VEGFR3 signaling has also been reported, and their effects in inhibiting tumor lymphangiogenesis and metastasis were remarkable.^309^ To target VEGFRs, blocking VEGFR3 signaling with VEGFR3 antagonist antibodies effectively inhibited angiogenesis, lymphangiogenesis, and tumor growth in an orthotopic spontaneous breast cancer metastasis model.^310^

Small-molecule inhibitors: The FDA has approved several small-molecule inhibitors of VEGFRs because they significantly prolong the survival of cancer patients. For instance, pazopanib is an orally available small-molecule inhibitor of VEGFRs, including VEGFR1, VEGFR2, and VEGFR3. Pazopanib showed favorable inhibitory effects on tumor growth, lymph node metastasis, and tumor lymphangiogenesis in an orthotopic colorectal cancer model.^311^ Sunitinib is a small-molecule inhibitor that targets VEGFRs, PDGFRs, and c-Kit.^312^ Sunitinib treatment markedly reduced pathological corneal lymphangiogenesis and angiogenesis.^313^ However, Dufies et al. reported that sunitinib treatment also induced lymphangiogenesis in the renal cell carcinoma context by activating VEGFC transcription and stabilizing VEGFC mRNA.^314^

Other inhibitors: Previous studies revealed that inhibition of VEGFC with specific siRNAs reduced the lymphangiogenesis in a murine mammary tumor model.^315,316^ Additionally, treatment with VEGFC siRNA effectively reduced lymphangiogenesis and significantly increased the survival rate of tumor-bearing mice. Fucoxanthin, a multifunctional natural non-pro-vitamin A carotenoid, has been shown to decrease tumor-associated lymphangiogenesis, indicating a potential anti-lymphangiogenic agent for use in cancer therapy.^317^ Shikonin, another natural compound isolated from the traditional Chinese medicinal herb Lithospermum erythrorhizon, inhibited lymphangiogenesis in a dose- and time-dependent manner.^318^ Curcumin, a natural dietary compound derived from turmeric, also inhibited lymphangiogenesis in vitro and in vivo by inhibiting the expression of VEGF receptors (Table 4).^319^

**Table 4:** Inhibitors of lymphangiogenesis and applications

Inhibitor name	Target of action	Application
Antibody drugs		
Bevacizumab	VEGF	Inhibition of Corneal inflammatory lymphangiogenesis Inhibition of melanoma-associated lymphangiogenesis
1E9	VEGFC	Inhibition of lymphangiogenesis in clear cell renal cell carcinoma
VGX-100	VEGFC	Suppression of lymphangiogenesis in corneal graft rejection response Inhibition of lymphangiogenesis in colorectal cancer
Single-chain fragment of VEGFC antibody	VEGFC	Blockage the lymphangiogenic activity of VEGFC
VEGFR3-immunoglobulin	VEGFC	Suppression of lung cancer-associated lymphangiogenesis
VEGFR3 antagonist antibody	VEGFR3	Suppression of lung cancer-associated lymphangiogenesis
MEDI3617	ANG2	Reduction of lung cancer-associated lymphangiogenesis
AZD5180	ANG2	Inhibition of lymphangiogenesis in infection-mediated inflammation
18E5	ANG2	Suppression of lymphangiogenesis in corneal graft rejection response
Small molecular inhibitors		
Pazopanib	VEGFR1, VEGFR2, VEGFR3	Inhibition of lymphangiogenesis in colorectal cancer
Sunitinib	VEGFR1, VEGFR2, VEGFR3, PDGFRs, c-Kit	Suppression of pathologic corneal lymphangiogenesis, Suppression of cancer-associated lymphangiogenesis
Synthesized preclinical candidate agents		
VEGFC siRNA	VEGFC	Suppression of lymphangiogenesis
ANG2 siRNA	ANG2	Suppression of pathologic corneal lymphangiogenesis
Inhibitors naturally existed in the host		
WNT1	VEGFC	Inhibition of melanoma-associated lymphangiogenesis
TSP-1	VEGFC	Suppression of corneal lymphangiogenesis Inhibition of lymphangiogenesis in mouse atherosclerotic aortic tissue
Semaphorins	Plexins and NRPs	Suppression of corneal lymphangiogenesis Suppression of HNSCC-associated lymphangiogenesis
IFN-γ		Promotion of LEC apoptosis
Other inhibitors		
Rapamycin	mTOR	Inhibition of cancer-associated lymphangiogenesis Suppression of lymphangiogenesis in corneal graft rejection response
Celecoxib	COX2	Suppression of lymphangiogenesis in breast cancer
Aspirin	COXs	Suppression of lymphangiogenesis in lung cancer
Phomaketide A	VEGFR3 PKCδ, eNOS	Inhibition of cancer-associated lymphangiogenesis
Fucoxanthin	-	Suppression of lymphangiogenesis in breast cancer
Shikonin	NF-κB	Inhibition of lymphangiogenesis in an in vitro model
Curcumin	VEGFR3	Suppression of cancer-associated lymphangiogenesis

*VEGF* vascular endothelial growth factor, *VEGFR* vascular endothelial growth factor receptor, *ANG* angiopoietin, *PDGFR* platelet-derived growth factor receptor, *NRP* neuropilin, *mTOR* mammalian target of rapamycin, *COX* cyclooxygenase, *PKC δ* protein kinase C δ, *eNOS* endothelial nitric oxide synthase

#### ANG

##### Agonistic effects of ANG

ANG and its receptors were first found to be important modulators in blood vessel growth, maturation, and stability. The two ANG receptors, called TIE1 and TIE2, were also reported to be expressed in LECs. After interaction with TIE on LECs, ANG induced the formation of TIE receptor complexes and then mediated cell behavior via activation of the PI3K/AKT pathway. AKT subsequently phosphorylated FOXO1, causing its nuclear exclusion and reducing the expression of FOXO1 downstream genes.^320^ Some findings revealed that ANG1 promoted lymphangiogenesis dependent on VEGFR3, indicating a difference in effects between ANG proteins.^321^ Interestingly, a recent study revealed that VEGFC induced ANG2 secretion from LECs and then stabilized VEGFR3 expression via the activation of ANG2/TIE/PI3K signaling, revealing cross-talk between the VEGFC and ANG2 signaling pathways.^322^ Additionally, ANG2 also functioned independent of its binding to TIE receptors. In this scenario, ANG2 bound β1 integrin and activated Src and FAK, leading to RhoA activation. The activation of RhoA led to the phosphorylation of the downstream effectors ROCK and formins, thereby regulating lymphangiogenesis.^323^

##### Targeted inhibitors of ANG signaling

Antibody drugs: Considering the effects of ANG2 on angiogenesis and lymphangiogenesis, several neutralizing antibodies for this signaling pathway have been designed and synthesized. These antibodies include trebananib, CVX-060, AMG 780, MEDI3617, Nesvacumab, Aflibercept, CVX-241, AZD5180, and 18E5. Among these neutralizing antibodies, MEDI3617, AZD5180, and 18E5 have been reported to show significant inhibitory effects on lymphangiogenesis.^324,325^

Small-molecule inhibitors: Regorafenib is a novel oral multikinase inhibitor that can inhibit various kinases, including VEGFRs, TIE, PDGFRs, c-Kit, BRAF, etc.^326^ In orthotopic colon tumor models, regorafenib treatment significantly decreased the density of lymphatic vessels in tumors.^327^

Other inhibitors: A previous study showed that siRNA-mediated ANG2 knockdown markedly inhibited corneal lymphangiogenesis.^76^ Recently, some multifunctional siRNA nanocapsules have been developed to deliver siRNA for targeting ANG in glioblastoma,^328,329^ which may provide a new direction for delivering siRNA for the treatment of abnormal lymphangiogenesis (Table 4).

#### Epidermal growth factor (EGF)

##### Agonistic effects of EGF

EGF belongs to a group of growth factors that specifically bind EGFR and activate EGFR signaling,^330^ which has been reported to participate in regulating skin lymphangiogenesis. EGF notably facilitated pathological lymphangiogenesis in melanoma, thus supporting the lymph node metastasis of melanoma.^331^ In summary, the effects of EGF on lymphangiogenesis have been relatively less studied than the effects of other factors and remain to be further investigated in the future.

##### Targeted inhibitors of EGF signaling

Antibody drugs: Some preclinical evidence has shown that monoclonal antibodies targeting EGF signaling could attenuate angiogenesis during cancer progression.^332,333^ However, evidence showing the inhibitory role of these monoclonal antibodies on lymphangiogenesis is lacking and further exploration is needed.

Small-molecule inhibitors: Afatinib, erlotinib, gefitinib, lapatinib, and vandetanib are representative small-molecule inhibitors of EGFR that prevent signal transduction after ligand-receptor binding and EGFR dimer formation. Among these drugs, lapatinib has been reported to attenuate tumor lymphangiogenesis and angiogenesis (Table 4).^334^

#### FGF2

FGF2 has been reported to exert a profound effect on lymphangiogenesis. In the mouse cornea, FGF2 promoted lymphangiogenesis in a dose-dependent manner and enhanced the secretion of VEGFC from vascular endothelial and perivascular cells to facilitate lymphangiogenesis.^335^ Additionally, FGF2 functions by binding the cell membrane LYVE1 with high affinity, which subsequently induces lymphangiogenesis.^336^

#### Hepatocyte growth factor (HGF)

HGF is a heparin-binding glycoprotein that was first shown to mediate liver regeneration. HGF functions by interacting with HGFR (also known as c-Met) to regulate lymphangiogenesis. Supplementation of HGF into the LEC culture medium promoted lymphangiogenesis, while inhibition of HGFR with an antagonist reduced the lymphangiogenesis.^337^

#### PDGF

PDGFs are secreted dimeric glycoprotein ligands with biological activities mediated by three forms of tyrosine kinase receptors encoded by two gene products, PDGFR-α and PDGFR-β.^338,339^ PDGF-BB functions as a lymphangiogenic factor and directly binds PDGFR-β expressed on LECs to induce lymph vessel growth.^340^

#### The insulin growth factor (IGF)

IGF signaling pathway is composed mainly of IGFs (IGF1 and IGF2), IGFRs (IGFR1, and IGFR2), and IGF-binding proteins (IGFBPs).^341,342^ IGFs bind IGFRs to initiate their effects, while IGFBPs interact with IGFs to modulate IGF stability and activity. IGFRs are expressed by LECs in both humans and mice. In vivo, administration of IGFs stimulated lymph vessel network expansion, while in vitro treatment with IGFs increased the lymphangiogenesis via activation of downstream ERK and PI3K/AKT signaling.^343^ In the future, the development of IGFs, IGFRs or IGFBPs antagonists may be an important approach for treating abnormal lymphangiogenesis.

#### WNT

Among the WNT family members, WNT5A and WNT5B have been reported to exert a positive effect on lymphangiogenesis.^344,345^ WNT5A regulates dermal lymphangiogenesis mainly through the noncanonical β-catenin-independent signaling pathway.^346^ Cancer cell-derived WNT5B modulated lymphangiogenesis and lymphatic permeability through the activation of both canonical and noncanonical WNT signaling pathways.^344^

#### EphrinB2

EphrinB2, a transmembrane ligand of the Eph receptor EphB4, controls cell migration and cytoskeletal organization in many different cell types and tissues.^347,348^ Evidence has shown that EphrinB2 and EphB4 are simultaneously expressed by endothelial cells and promote sprouting behavior to induce angiogenesis and lymphangiogenesis.^349,350^ Mechanistically, the binding of EphrinB2 to EphB4 promoted the activation of the small GTPases Rac1, AKT, and ERK and promoted VEGFC/VEGFR3 downstream signaling. In capillary lymphatic vessels of adult mouse corneas, the EphrinB2/EphB4 axis functioned in the formation and maintenance of funnel-shaped valves, indicating that this axis might be an ideal target for regulating corneal lymphangiogenesis.^351^

#### Adrenomedullin

Adrenomedullin, a well-known vasodilator, is also an important regulator of lymphangiogenesis. Adrenomedullin functions by binding its specific receptor, CALCRL. Ablation of Adrenomedullin/CALCRL signaling impedes the activation of ERK signaling, leading to the formation of abnormal jugular lymphatic vessels.^63^ Blockade of Adrenomedullin/CALCRL signaling after normal lymphatic vessel formation resulted in impaired permeability and function in intestinal, corneal, and dermal lymphatic vessels.^352^ In several types of cancers, Adrenomedullin significantly increased tumor-associated lymphangiogenesis.^353,354^

#### Bioactive lipids

**Fig. 14:**
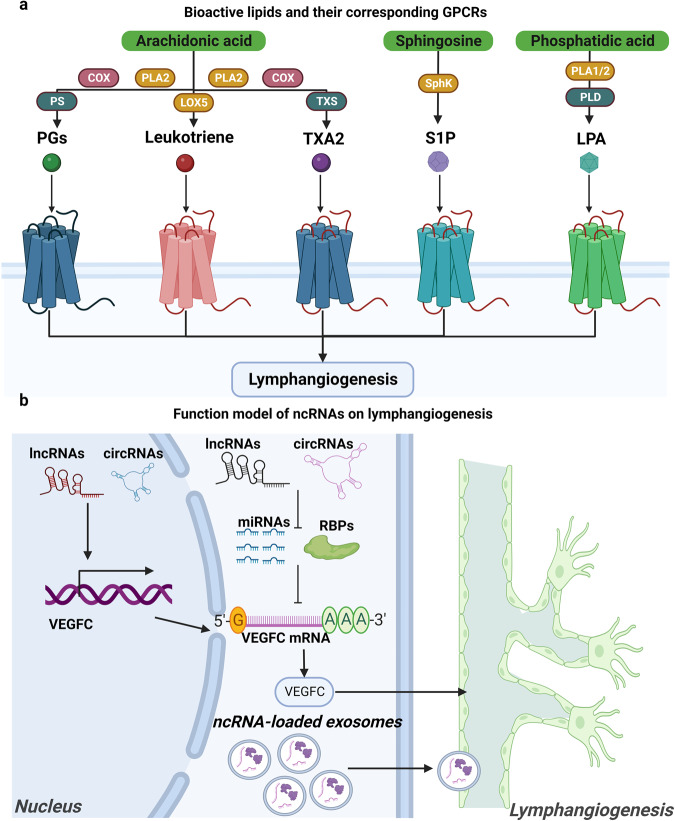
The role of bioactive lipids and ncRNAs on lymphangiogenesis. **a** The bioactive lipids derived from the metabolism of arachidonic acid, Sphingosine, phosphatidic acid could regulate lymphangiogenesis through binding their specific GPCRs; **b** The function model of ncRNAs on lymphangiogenesis. Some ncRNAs were reported to have evident role on lyphangiogenesis, especially in tumor associated-lymphangiogenesis. These ncRNAs could be potential therapeutic targets for controlling abnormal lymphangiogenesis in cancer. GPCRs G Protein-Coupled Receptors, COX cyclooxygenase, PLA2 phospholipase A2, PS prostanoid synthases, TXA thromboxane, LOX5 5-lipoxygenase, S1P sphingosine 1-phosphate, LPA lysophosphatidic acid, ncRNAs noncoding RNAs, lncRNAs long noncoding RNAs, circRNAs circular RNAs, RBPs, RNA binding proteins. Created with BioRender.com

Bioactive lipids are important regulators in the body; they include 1) arachidonic acid (AA) and its metabolites, such as prostaglandins (PGs), thromboxane (TXA), and leukotrienes (LTs); 2) S1P, a metabolic product of sphingolipids; and 3) lysophosphatidic acid (LPA). These bioactive lipids have been shown to regulate lymphangiogenesis under certain pathological conditions. We summarize the function of these common bioactive lipids in lymphangiogenesis (Fig. 14).

##### PGs

PGs are common metabolites of AA generate through the action of phospholipase A2, cyclooxygenases (COXs), and the respective prostanoid synthases. PGs function by binding their specific receptors, which are often transmembrane G protein-coupled receptors.^355,356^ In an LPS-induced model of inflammation, COX2-derived PGs enhanced lymphangiogenesis.^357^ Similarly, in acute colitis induced by DSS, PGE2/PGE receptor (EP) 4 signaling stimulates lymphangiogenesis to repair damaged mucosa. PGE2-EP4 activation on tumor cells and TAMs increased the amount of VEGFC and VEGFD secreted from these cells and then stimulated LEC lymphangiogenesis.^358^ Tumor-derived PGE2 also directly acted on LECs to activate EP4 and VEGFR3 and ultimately induced lymphangiogenesis.^359^

##### TXA2

TXA2 is an unstable metabolite of AA produced by the reciprocal action of COX and TX synthase.^360,361^ TXA2 plays a regulatory role by binding thromboxane prostanoid (TP), a transmembrane G protein-coupled receptor. TXA2-TP signaling facilitates lymphangiogenesis by acting on macrophages and T cells during inflammation. TP-positive macrophages and T cells infiltrate inflamed tissue and produce VEGFC and VEGFD after stimulation with TXA2. These macrophage- and T-cell-derived VEGFC/D promoted lymphangiogenesis and drainage function in mice with inflammatory stress.^362^

##### LTs

LTs constitute a class of bioactive lipids derived from AA by 5-lipoxygenase (LOX5) and LOX5-activating protein.^363,364^ LTs also function by binding G protein-coupled receptors. Two G protein-coupled receptors, BLT1 and BLT2, are receptors for LTB4 and are expressed in macrophages, leukocytes, epidermal keratinocytes, and epithelial cells. Evidence from a lymphedema model showed that macrophages are important sources of LTB4 and that inhibition of LTB4 signaling effectively prevents edema.^365,366^

##### S1P

S1P is the phosphorylated form of sphingosine that is formed through the action of sphingosine kinase 1 (SphK1) and SphK2. S1P not only functions intracellularly as a second messenger, but also can be exported extracellularly to serve as a ligand for a family of S1P receptors.^367^ S1P promotes lymphangiogenesis both in vitro and in vivo through the S1P/Gi/phospholipase C/Ca^2+^ signaling pathways.^368^ In breast cancer, SphK1-mediated S1P production induced lymphangiogenesis, indicating that SphK1-S1P signaling may be a potential therapeutic target for controlling lymphangiogenesis.^369^

##### LPA

LPA is a low-molecular-weight lipid growth factor that functions by binding to Edg family members.^370^ LPA is generated through enzymatic cleavage of membrane phosphatidic acid. A series of studies have revealed that LPA promoted lymphangiogenesis by increasing the expression of the important prolymphangiogenic factor VEGFC in targeted cells such as tumor cells and endothelial cells.^371,372^

#### NcRNA

NcRNA transcripts constitute a recently described type of RNA that has been extensively explored in recent decades. Some ncRNAs have been reported to play a clear role in lymphangiogenesis, especially in tumor-associated lymphangiogenesis (Fig. 14).

##### LncRNAs

LncRNAs are RNA transcripts longer than 200 bases with low protein-coding potential.^373^ Some lncRNAs play a prolymphangiogenic role intracellularly to enhance the expression of VEGFC, thereby promoting lymphangiogenesis.^374,375^ Cancer cells also deliver lncRNA-containing exosomes to LECs. The lncRNA cargo internalized by LECs induced lymphangiogenesis both in vitro and in vivo.^376^ These pieces of evidence implicate lncRNAs as important therapeutic targets for controlling abnormal lymphangiogenesis in patients with cancer (Fig. 14).

##### CircRNAs

CircRNAs represent a type of novel ncRNA with a covalently circular structure generated from the splicing of pre-mRNAs and interlacing of the 5′ caps and 3′ poly-A tails and are specific messenger RNAs.^377,378^ The role of circRNAs in lymphangiogenesis was investigated recently. For instance, circEHBP1 served as a miRNA sponge for miR-130a-3p to regulate the TGFβR1/VEGFD axis, leading to increased levels of VEGFD and lymph vessel density in the context of bladder cancer.^203^ Additionally, some circRNAs are transported from cancer cells to LECs via exosomes. In this manner, cancer cell-derived circRNAs can directly function as intra-LECs to contribute to lymphangiogenesis.^379^

### Clinical trials of treatments for lymphangiogenesis-related diseases

We conducted an electronic search for relevant clinical trials of lymphangiogenesis-related diseases in PubMed. Additionally, relevant clinical trial registration sites, such as ClinicalTrials.gov and Netherlands Trial Registry, were comprehensively examined. Literature retrieval was performed in duplicate by two independent reviewers. A total of ten published clinical trials were included in the analysis, and among these, seven studies mainly examined the effects of different interventions on lymphangiogenesis-related diseases in human samples, one study focused on evaluating stem cell therapy in lymphedema,^380^ one study focused on circulating angiogenic factors in pulmonary tuberculosis in which angiogenesis and lymphangiogenesis were classical features,^381^ and one study mainly examined whether PDPN expression correlated with sentinel lymph node metastasis in early squamous cell carcinomas of the oral cavity and oropharynx.^382^ The characteristics of these studies are listed in Supplementary Table 1.

The ten selected clinical trials had enrolled participants with eight different diseases and conditions. More than three studies enrolled participants with breast cancer, including HER2-negative breast cancer and lymph node-positive breast cancer.^383–385^ A few studies recruited participants with other diseases, including lung adenocarcinoma,^386^ early squamous cell carcinomas of the oral cavity and oropharynx, Type 1 diabetes,^252^ tongue cancer,^387^ lower limb lymphedema, lymphangioleiomyomatosis (LAM)^388^ and pulmonary tuberculosis. The study with the largest number of participants enrolled patients with centrally located squamous cell carcinoma with cavitary features, and those with brain metastases that were uncontrolled or controlled for less than 2 months were excluded (*n* = 440). The patients were randomly assigned in a 2-to-1 ratio to receive either 12 mg/day of anlotinib (*n* = 294) or a matched placebo (*n* = 146).

With regard to the intervention/comparison in the studies, over half of the studies (*n* = 5) focused on drug (anlotinib treatment for lung adenocarcinoma) and stem cell therapy (using bone marrow-derived mononuclear cells in the treatment of lower limb lymphedema), and 1 study focused on dietary interventions such as salt intake. Salt-sensitive blood pressure increases in Type 1 diabetes patients is accompanied by disturbed skin macrophage influx and lymphatic dilation. In an evaluation of the medical treatment of patients with breast cancer, two clinical trials demonstrated that treatment with sunitinib showed favorable effects on tumor vessel modulation and lymphangiogenesis and significantly decreased lymphatic vessel density, as assessed via immunohistochemistry. To assess a lung adenocarcinoma medical treatment, one clinical trial demonstrated that anlotinib suppressed lymphangiogenesis and lymphatic metastasis through a process potentially involving VEGFR3 signaling. Another clinical trial showed that in patients with lymphangioleiomyomatosis, sirolimus stabilized lung function and reduced the level of serum VEGFD, which is a lymphangiogenic growth factor implicated in the pathophysiology of LAM and was associated with a reduction in symptoms and improvement in quality of life. A study of patients with chronic lymphedema showed that cell therapy led to reduced limb circumference and increased pain relief and improved walking ability compared with the results in the control group.

Approximately 40.0% (*n* = 4) of published clinical trials explored the effects of lymphangiogenesis activators under pathological conditions. One study showed that HER2/neu expression correlated with VEGFC and lymphangiogenesis in lymph node-positive breast cancer patients. Another study demonstrated that pulmonary tuberculosis was associated with elevated circulating levels of VEGFA, VEGFC, and VEGFR2, and angiogenesis and lymphangiogenesis were shown to be classical features of granuloma formation. Recently, the expression of PDPN in cancer cells was demonstrated to promote tumor cell motility and tumor lymphangiogenesis in vitro, and one supplementary clinical trial showed that PDPN expression correlated with sentinel lymph node metastasis in early squamous cell carcinomas of the oral cavity and oropharynx. NRP2 plays an important role in regulating lymphangiogenesis, and findings from a clinical trial for patients with early-stage tongue cancer showed that cytoplasmic NRP2 was associated with metastasis and a poor prognosis.

Although several published clinical trials showed consistent findings and most studies suggested that drug interventions targeting activators or inhibitors suppressed lymphangiogenesis-related signaling, lymphangiogenesis activators played an important role in attenuating the pathological state. Given the substantial impact of lymphangiogenesis progression on health and disease, a larger sample, longer intervention period, and multicenter clinical trials are needed to examine the safety and efficacy of targeted interventions.

## Conclusion

Since the discovery of lymphatic vessels exists in organisms, an increasing number of studies have revealed the mapping of lymphatic vessel anatomy and development. In this review, we described the general lymphatic vessel development and regulatory signaling pathways. Meanwhile, organ-specific lymphatic vessel development has also shown heterogeneous processes and signaling regulation catering to tissue development and physiological functions. However, the lymphangiogenic regulatory landscape remains a puzzle. Therefore, further mechanism studies are required to determine the phenotypic differences of lymphatic vessels in diverse developmental stages and tissues, including the key transcriptome and epigenome function. The robust lymph absorption and transport capacity of lymphatic vessels contribute to fluid homeostasis. In addition, lymphatic vessels directly or indirectly participate in immunosurveillance and immune response within diverse tissues and organs.

Lymphatic vessels are actively involved in multiple diseases in humans and alleviate or exacerbate local pathological progression through lymphangiogenesis. Notably, many studies have great interest in exploring lymphangiogenesis in tumors, which actively participates in the process of lymph node metastasis and affects the tumor prognosis and treatment. Moreover, LECs are involve in the regulation of tumor-specific immune response by affecting migration, function, and survival of immune cells. Exploration of crosstalk between LECs and immune cells may be a powerful target to enhance immunosurveillance for tumors in humans. In the future, exploration of the specific markers for tumor-associated lymphangiogenesis assists clinical tracing of metastatic lymph nodes, which may provide the specification for accurate lymph node resection. Targeting lymphangiogenesis is a potential and powerful intervention for restoring lymphatic vessel function and improving disease treatment. Based on these, we systematically review the lymphangiogenic signaling pathways, with specific inhibitors known till now. Moreover, multi-center clinical trials are being carried out to further demonstrate the feasibility and broad prospects of targeting abnormal lymphangiogenesis in a variety of diseases.

In conclusion, we have summarized the heterogeneous characteristics and functions of lymphatic vessels in health and disease conditions and expect to provide comprehensive knowledge for future research to realize translational therapy.

## Supplementary information

Supplemental Table 1
