# Supplementary material for: Lymphatic vessel: Origin, heterogeneity, biological functions and therapeutic targets
Source: Signal Transduct Target Ther. 2024 Jan 3;9:9. doi: 10.1038/s41392-023-01723-x (PMC10764842; doi:10.1038/s41392-023-01723-x)
Supplement: Supplementary file 1 — Supplemental Table 1 [file 41392_2023_1723_MOESM1_ESM.docx]

**Supplemental Table 1.** Summary of published clinical trials on lymphangiogenesis-related diseases

| **Trial** | **Year** | **Phase** | **Participants** | **Intervention/**  **comparison** | **Sample size of intervention/**  **comparison** | **Outcome** | **Main findings** |
| --- | --- | --- | --- | --- | --- | --- | --- |
| NCT01176799 | 2016 | Ⅰb/Ⅱ | Treatment-naive breast cancer patients | Sunitinib 12.5 mg daily for 7 days before each chemotherapy/ Chemotherapy plus sunitinib/Chemotherapy | 9/25/24 | Phase Ib (n=9) was established as RP2D. In phase II, patients receiving chemotherapy  plus sunitinib (n=24) had a higher incidence of chemotherapy dose delays, and significantly increased VNI and decreased LVD on immunohistochemistry. | The addition of sunitinib induced compelling pharmacodynamic evidence of vascular normalization. |
| NCT02790580 | 2016 | Ⅱ | HER2-negative breast  cancer patients | Sunitinib/Bevacizumab  pre-treatment with chemotherapy | 38/24 | Sunitinib led to a significant increase in VNI post-C1 and -C4  along with decrease in LVD post- C1. | Sunitinib showed a greater effect on lymphangiogenesis suggesting that its administration prior to chemotherapy might result in improved drug delivery. |
| NCT02388919 | 2015 | Ⅲ | Advanced lung  adenocarcinoma patients | Anlotinib/Placebo | 294/146 | Anlotinib suppressed the rate of new metastatic lesions (31.82% in the placebo arm  and 18.18% in the anlotinib arm) in patients with advanced lung adenocarcinoma. | Anlotinib may be beneficial for treatment in avoiding lymphangiogenesis and distant  lymphatic metastasis in lung adenocarcinoma. |
| NCT00414648 | 2011 | Ⅲ | LAM patients | Sirolimus/Placebo | 46/43 | Sirolimus had an improvement from baseline to 12 months in measures of forced vital capacity, functional residual capacity, serum VEGFD level, quality of life and functional performance. | Sirolimus stabilized lung function, reduced serum VEGFD level and symptoms and improved quality of LAM patients’ life. |

**Supplemental Table 1.** Continued

| NTR4095,  NTR4788 | 2020 | N/A | Type 1 Diabetes patients | Type 1 diabetes patients/ Healthy controls | 8/12 | Type 1 diabetes patients (n = 8) showed a salt-sensitive BP  increase, whereas healthy controls (n = 12) showed increased skin CD163^+^ and HLA-DR^+^ macrophages and dilation of skin lymphatic vessels. | Salt sensitivity in type 1 diabetes  patients cannot be explained by the classical concept of extracellular fluid volume expansion. Rather, the study opens up a potential role for macrophages and the lymphatic  system. |
| --- | --- | --- | --- | --- | --- | --- | --- |
| N/A | 2018 | N/A | Chronic lymphedema patients | Stem cell therapy/  Compression therapy | 20/20 | Biopsy examine showed a marked increase in the number of capillary lymphatic vessels in stem cell therapy group. | Stem cell therapy can achieve an improvement in limb circumference as well as pain relief and improvement in walking ability in patients with chronic lymphedema. |
| N/A | 2017 | N/A | cT1-2N0M0 tongue SCC patients | Prophylactic  neck dissection/ Watchful waiting | 50/50 | Cytoplasmic NRP2 overexpression predicted regional lymph node metastasis with sensitivity and specificity of 90.3% and 42.1%. | Cytoplasmic NRP2 overexpression may be a diagnostic and prognostic marker for early tongue SCC. |
| NCT01154959 | 2016 | Ⅲ | Active PTB/LTB/NTB patients | Examined the circulating levels of angiogenic factors in individuals with PTB/LTB/NTB | 42/44/44 | Circulating levels of VEGFA, VEGFC and VEGFR2 were significantly higher in PTB with bilateral and/or cavitary disease. The circulating levels of all the angiogenic factors reduced following successful chemotherapy. | PTB is associated with elevated levels of circulating angiogenic factors, possibly reflecting vascular and endothelial dysfunction. |
| N/A | 2011 | N/A | Early HNSCC patients | PDPN expression | 120 | 29 of 120 primary HNSCC showed PDPN expression, which was correlated with SLN metastasis and remained a significant predictor for lymph node status. | PDPN expression is associated with lymph node metastasis *in vivo.* |

**Supplemental Table 1.** Continued

| N/A | 2010 | N/A | Stage II node-positive invasive breast cancer patients | VEGFC, LMVD, LVI and HER2/ neu expression | 150 | Cases with 3+ HER2/neu protein expression showed a significantly stronger VEGFC expression than all other cases. | Inhibiting HER2/neu may suppress tumor progression by blocking VEGFC-mediated tumor cell proliferation and lymph node metastasis. |
| --- | --- | --- | --- | --- | --- | --- | --- |

*LAM* lymphangioleiomyomatosis, *VEGF* vascular endothelial growth factor, *VNI* vascular normalization index, *C1* cycle 1, *C4* cycle 4, *LVD* lymphatic vessel density, *RP2D* recommended phase II dose, *LMVD* lymphatic microvessel density, *LVI* lymphovascular invasion, *SCC* squamous cell carcinoma, *NRP2* neuropilin 2, *PTB* pulmonary tuberculosis, *LTB* latent tuberculosis, *NTB* individuals with no tuberculosis, *HNSCC* head and neck squamous cell carcinoma, *PDPN* podoplanin, *SLN* sentinel lymph node.
